# Association of Daytime-Only, Nighttime-Only, and Compound Heat Waves With Preterm Birth by Urban-Rural Area and Regional Socioeconomic Status in China

**DOI:** 10.1001/jamanetworkopen.2023.26987

**Published:** 2023-08-11

**Authors:** Yafei Guo, Peiran Chen, Yanxia Xie, Yanping Wang, Yi Mu, Ruobing Zhou, Yanlin Niu, Xiaoming Shi, Jun Zhu, Juan Liang, Qiyong Liu

**Affiliations:** 1 National Key Laboratory of Intelligent Tracking and Forecasting for Infectious Diseases, National Institute for Communicable Disease Control and Prevention, Chinese Center for Disease Control and Prevention, Beijing, China; 2 Chinese Center for Disease Control and Prevention Key Laboratory of Environment and Population Health, National Institute of Environmental Health, Chinese Center for Disease Control and Prevention, Beijing, China; 3 National Office for Maternal and Child Health Surveillance of China, West China Second University Hospital, Sichuan University, Chengdu, China; 4 Key Laboratory of Birth Defects and Related Diseases of Women and Children (Sichuan University), Ministry of Education, Chengdu, China; 5 Department of Health, Ethics and Society, Maastricht University, Maastricht, the Netherlands; 6 Institute for Nutrition and Food Hygiene, Beijing Center for Disease Prevention and Control, Beijing, China; 7 Chinese Center for Disease Control and Prevention, Beijing, China

## Abstract

**Importance:**

Associations between heat waves and preterm birth (PTB) have been reported. However, associations of daytime-only, nighttime-only, and compound heat waves with PTB have yet to be explored at a national level. Furthermore, possible heterogeneity across urban-rural communities with different socioeconomic statuses needs to be explored.

**Objective:**

To examine the association between daytime-only, nighttime-only, and compound heat waves and PTB in China and to find variations between urban and rural regions.

**Design, Setting, and Participants:**

This case-crossover study used nationwide representative birth data between January 1, 2012, and December 31, 2019, from China’s National Maternal Near Miss Surveillance System. This multisite study covered 30 provinces in China and ensured the representation of urban and rural populations across 3 socioeconomic regions. Singleton live births delivered in the warm seasons from April to October during the study period were included. Exclusion criteria consisted of gestational age younger than 20 or older than 45 weeks, maternal ages younger than 13 or older than 50 years, conception dates earlier than 20 weeks before January 1, 2012, and later than 45 weeks before December 31, 2019, and an inconsistent combination of birthweight and gestational age according to growth standard curves of Chinese newborns. Data were analyzed from September 10, 2021, to April 25, 2023.

**Exposures:**

Eighteen definitions of heat waves by 3 distinct types, including daytime only (only daily maximum temperature exceeds thresholds), nighttime only (only daily minimum temperature exceeds thresholds), and compound (both daily maximum and minimum temperature exceeds thresholds) heat waves, and 6 indexes, including 75th percentile of daily temperature thresholds for 2 or more (75th-D2), 3 or more (75th-D3), or 4 or more (75th-D4) consecutive days and 90th percentile of daily temperature thresholds for 2 or more (90th-D2), 3 or more (90th-D3), and 4 or more (90th-D4) consecutive days.

**Main Outcomes and Measures:**

Preterm births with less than 37 completed weeks of gestation.

**Results:**

Among the 5 446 088 singleton births in the final analytic sample (maternal mean [SD] age, 28.8 [4.8] years), 310 384 were PTBs (maternal mean [SD] age, 29.5 [5.5] years). Compared with unexposed women, exposure of pregnant women to compound heat waves in the last week before delivery was associated with higher risk for PTB, with the adjusted odds ratios (AORs) ranging from 1.02 (95% CI, 1.00-1.03) to 1.04 (95% CI, 1.01-1.07) in 6 indexes. For daytime-only heat wave exposures, AORs ranged from 1.03 (95% CI, 1.01-1.05) to 1.04 (95% CI, 1.01-1.08) in the 75th-D4, 90th-D2, 90th-D3, and 90th-D4 indexes. Such associations varied by rural (AOR range, 1.05 [95% CI, 1.01-1.09] to 1.09 [95% CI, 1.04-1.14]) and urban (AOR range, 1.00 [95% CI, 0.98-1.02] to 1.01 [95% CI, 0.99-1.04]) regions during exposure to daytime-only heat waves in the 75th-D3 and 90th-D3 indexes.

**Conclusions and Relevance:**

In this case-crossover study, exposure to compound and daytime-only heat waves in the last week before delivery were associated with PTB, particularly for pregnant women in rural regions exposed to daytime-only heat waves. These findings suggest that tailored urban-rural preventive measures may improve maternal health in the context of climate change.

## Key Points

**Question:**

Is exposure to daytime-only, nighttime-only, and compound heat waves associated with preterm birth among Chinese women with singleton pregnancies, and does the association vary across urban-rural and socioeconomic regions?

**Findings:**

This case-crossover study linked nationwide data representing 5 446 088 singleton pregnancies in the warm season from 2012 to 2019. Exposure to compound and daytime-only heat waves was associated with preterm birth, particularly for rural mothers exposed to daytime-only heat waves.

**Meaning:**

These findings suggest that protecting pregnant women from compound and daytime-only heat waves may improve heat-related preterm birth in China through targeted measures.

## Teaser

This case-crossover study estimates the association between exposure of pregnant women to heat waves in the last week before delivery and preterm birth and examines urban-rural and socioeconomic regional variations in heat wave–related preterm births in China.

## Introduction

Several studies have reported associations between exposure to high temperatures in pregnancy and risk of preterm birth (PTB).^1,2,3,4,5,6,7^ A recent systematic review and meta-analysis^8^ reported higher odds of PTB during heat waves, which also highlighted a link between maternal socioeconomic status (SES) and risk of PTB during heat waves. Studies also found that associations between exposure to heat and PTB were stronger in low SES groups, such as economically and educationally disadvantaged groups.^6,8^

Given the lack of a universal definition of a heat wave, defining heat waves are also related to the risk of PTB.^9^ Many published epidemiological studies only used daytime heat exposure in their studies.^1,2,6,9^ Several recent studies have considered the influence of heat waves with both daily maximum (Tmax) and minimum temperatures (Tmin), emphasizing varied human thermoregulatory responses to daytime and nighttime heat waves,^10^ namely: daytime-only, nighttime-only, and compound heat waves.^11^ Hence, it is worthwhile to adopt these new heat wave classifications to examine their associations with risks for PTB. This is particularly important in China given the various regions with different climate characteristics and socioeconomic development levels.^10,12^

In addition to maternal SES, studies have also suggested socioeconomic regional variations in heat-related PTB.^13,14,15,16^ Meanwhile, research findings about urban-rural disparities in heat-related PTB are limited and inconsistent,^9,14^ with little such evidence available in China. It is important to assess urban-rural and socioeconomic region–specific associations between heat waves and PTB, because the findings could provide critical evidence for developing heat adaptation policies to guide clinical practice, to undertake community health education, and to prevent PTB. Using a nationwide maternal surveillance database, this study aims to estimate the association between exposure to heat waves in the last week before delivery and preterm birth and to examine urban-rural and socioeconomic regional variations in heat wave–related PTB.

## Methods

### Study Population

**Figure 1.**
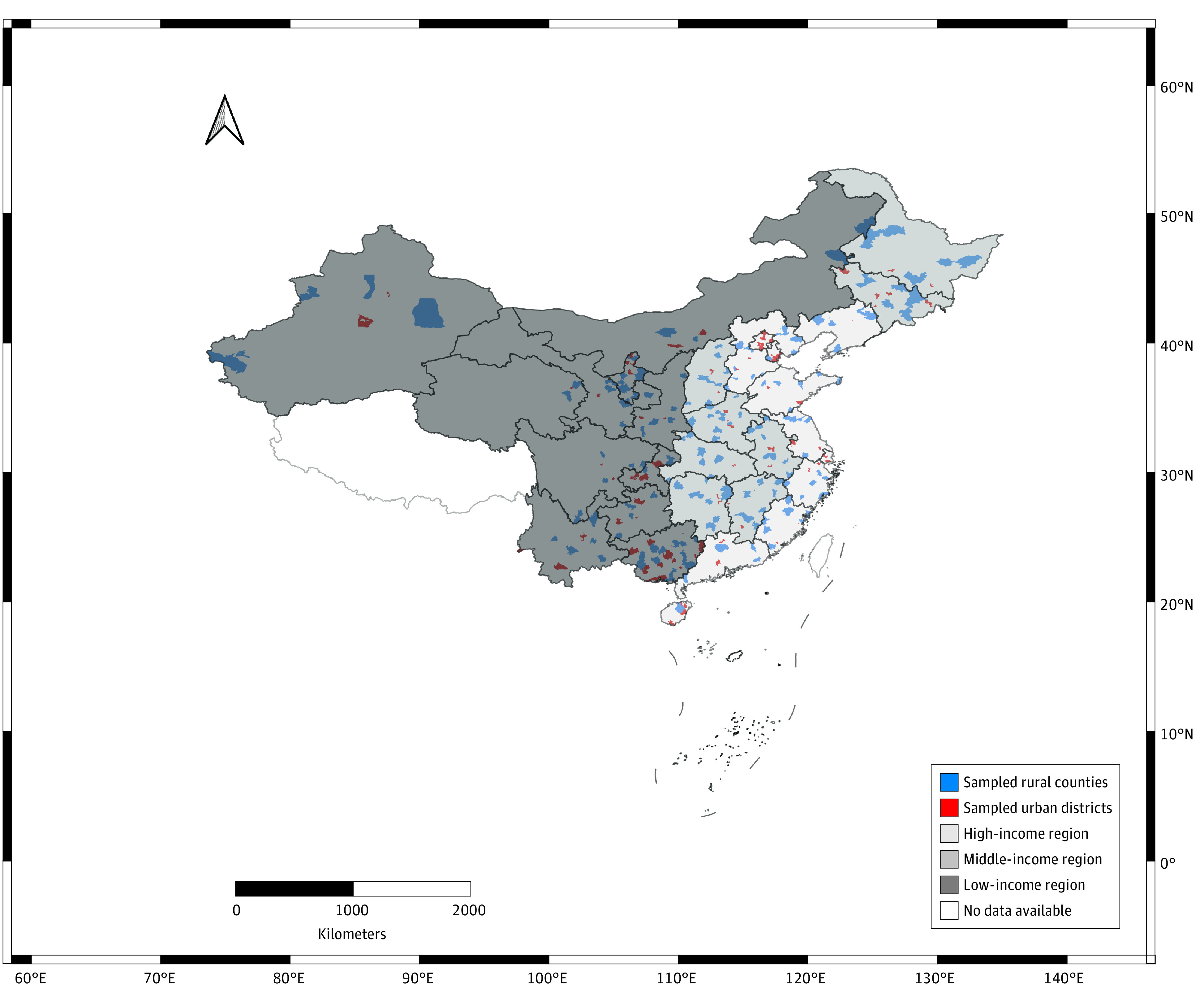
Distribution of Sampled Rural Counties, Urban Districts, and 3 Subnational Socioeconomic Regions in China

We obtained data on live births for this case-crossover study between January 1, 2012, and December 31, 2019, from China’s National Maternal Near Miss Surveillance System (NMNMSS). Data consisted of 438 sampled health facilities in 325 counties or districts across China, including 170 rural counties and 155 urban districts (Figure 1 and eMethods and eTable 1 in Supplement 1).^17,18,19^ The NMNMSS was approved by the Ethics Committee of West China Second University Hospital, Sichuan University, and followed the tenets of the Declaration of Helsinki. In addition to establishing the NMNMSS, the ethical approval also permitted the use of data for subsequent studies (including the present study) on maternal health from the NMNMSS and waived informed consent because of using previously collected administrative surveillance data. This study followed the Strengthening the Reporting of Observational Studies in Epidemiology (STROBE) reporting guideline.

We included singleton live births between January 1, 2012, and December 31, 2019 (N = 10 796 749). Exclusion criteria consisted of gestational age younger than 20 weeks or older than 45 weeks, maternal ages younger than 13 years or older than 50 years, conception dates earlier than 20 weeks before January 1, 2012, and later than 45 weeks before December 31, 2019, and an inconsistent combination of birthweight and gestational age according to growth standard curves of Chinese newborns. After applying these 4 exclusion criteria (eFigure 1 in Supplement 1),^20,21,22,23^ we included 9 189 216 singleton births and extracted 5 446 088 births in the warm season from April to October in the final analyses. We set the warm season considering diverse climate type in this national study.^24^

We assigned eligible birth records with county-level rurality and region-level SES by the addresses of delivery health facilities for each pregnant woman, as the residential addresses were unavailable in the NMNMSS. According to China’s National Health Statistical Yearbook,^25^ we classified rurality (rural and urban) and 3 regions (Eastern, Central, and Western) (eMethods in Supplement 1). Eastern, Central, and Western regions represented high-, middle- and low-income regions. We also classified county-level rurality by calculating the mean grid annual gross domestic product (GDP), GDP per capita, population, and warm season normalized difference vegetation index (NDVI) within an urban district or rural county (eMethods in Supplement 1).^26,27,28^ The NDVI were generated from satellite data products,^28^ which provides quantified reflectance measurements for vegetation by remote sensing images. The index ranges from −1 to +1, with higher NDVI values indicating more dense vegetation. We counted the warm season NDVI for April to October 2015, the middle year of our study period (eMethods in Supplement 1).^26,27,28^

### Outcome Definition

We defined PTB as births with less than 37 completed weeks of gestation.^29^ We calculated gestational age based on the last menstrual period.

### Exposure Assessment

Daily Tmax and Tmin were obtained from data set CN05.1 from the National Climate Center of China Meteorological Administration, which has been described elsewhere in further detail.^30,31^ This daily meteorological data set with a resolution of 0.25° latitude by 0.25° longitude was based on the interpolation from over 2400 observational stations in China and included daily Tmin and Tmax in degrees Celsius. Daily relative humidity was downloaded from the fifth-generation European Centre for Medium-Range Weather Forecasts with a resolution of 0.5° latitude by 0.5° longitude. We obtained daily fine particulate matter levels with an aerodynamic diameter less than or equal to 2.5 μm (PM_2.5_) and ozone levels from the Tracking Air Pollution in China data set with a resolution of 0.1° latitude by 0.1° longitude.^32,33,34^ Daily ozone was only available since January 1, 2013. Depending on the results of maternal care surveys, the distance from the residence to the hospital address available in the NMNMSS was substantial for pregnant women choosing their delivery hospitals, normally within 25 km.^35^ We collected and calculated the mean grid temperature and air pollutants from a zone with a 25-km radius around each woman’s delivery health facility address to assign environmental exposure.

### Definition of 3 Types and 6 Indexes of Heat Waves

We defined 3 types of heat waves and 6 indexes based on previous studies of hot weather events.^1,2,10,36,37^ A heat wave is defined as a period of at least 2, 3, or 4 consecutive days above the daily temperature threshold.^1,2,37^ We calculated daily thresholds within each health facility’s 25-km radius domain based on 30 years in the reference period, from 1981 through 2010, the recent climate state period. The following 3 types of heat waves were then defined: (1) daytime-only heat wave, which contains only thresholds of daily Tmax; (2) nighttime-only heat wave, which contains only thresholds of daily Tmin; and (3) compound heat wave, which contains thresholds of both daily Tmax and Tmin. We then defined the following 6 indexes: (1) 75th percentile of daily temperature thresholds with 2 or more consecutive days (75th-D2); (2) 75th percentile of daily temperature thresholds with 3 or more consecutive days (75th-D3); (3) 75th percentile of daily temperature thresholds with 4 or more consecutive days (75th-D4); (4) 90th percentile of daily temperature thresholds with 2 or more consecutive days (90th-D2); (5) 90th percentile of daily temperature thresholds with 3 or more consecutive days (90th-D3); and (6) 90th percentile of daily temperature thresholds with 4 or more consecutive days (90th-D4). We calculated the daily binary variables to identify heat wave days with 18 definitions (3 types in 6 indexes) of heat waves in each health facility’s 25-km radius domain during 2012 to 2019 (eMethods in Supplement 1).

### Study Design 

We used a space-time–stratified case-crossover design^38^ in this multisite study to estimate the association between heat wave events and PTB, with site-specific year and calendar month as the fixed time and space stratum. This design used each pregnant woman as her own control and compared exposure between case day and control days. The delivery date of PTB was the case day. Control days comprised the same day of the week in the calendar month of PTB onset; each case day would have 3 or 4 control days (eTable 2 in Supplement 1). This design controlled for long-term trends, seasonality, effects of the day of the week, and overlap effects.^39,40^

### Statistical Analysis

Data were analyzed from September 10, 2021, to April 25, 2023. We used conditional logistic regression models to assess the association between heat wave events and PTB, controlling time-invariant individual level confounders (eg, maternal age and educational attainment).^41^ Heat wave day was assigned in the model as the independent binary variable (yes or no), and the indicator of case-control status (1 or 0) served as the outcome measure.^41^ Given recent evidence on the acute effects of PM_2.5_ on PTB in China and inconsistent evidence on ozone,^1,41,42^ we only considered PM_2.5_ as a covariate in our main models to adjust potential confounding from air pollutants. Meanwhile, we also include relative humidity as a covariate to account for its potential influence on the association.^2^ We adjusted all models for moving mean lag period for the previous 6 days (lag6) of relative humidity and PM_2.5_ in the last gestational week, calculated across the time window,^42,43^ as continuous variables using a natural cubic spline with 3 *df* to control the nonlinear association between relative humidity or PM_2.5_^41^ and PTB. Models were performed one by one for each heat wave definition of 3 types and 6 indexes. We applied 1-stage analysis in multilocation to fit the space-time–stratified case-crossover design. We examined lag effects of the last week before delivery, with 7 single lag days (lag0, lag1, lag2, lag3, lag4, lag5, and lag6). We then estimated the overall odds ratio (OR) with the maximum points estimation in the 7 lag days under each heat wave definition. In sensitivity analyses, we adjusted ozone and changed the *df* of the natural cubic spline for relative humidity and PM_2.5_ to assess the robustness of the models.

We examined whether the associations varied by rural and urban regions and subnational socioeconomic regions using fully stratified models. In all subgroup analyses, we used the same lag as the overall estimation. We implemented a 2-sample test to evaluate whether associations differed significantly across subgroups (a 2-sided *P* < .05 for 2 samples and *P* < .025 for triple-group comparisons).^44,45^ For example, to determine whether the risk estimates were significantly different between rural and urban, we have the following equation:



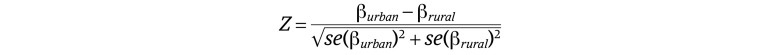



We also examined the variation in models with an interaction term of heat wave exposure variable and the category variable of urban or rural region or subnational socioeconomic region. We tested the significance of the interaction term (a 2-sided *P* < .05). All analyses were conducted in R, version 4.1.1 (R Project for Statistical Computing). We used the survival (version 3.2.11) and splines (version 4.1.1) packages for conditional logistic regression analysis.

## Results

A total of 5 446 088 singleton births in the warm season (59.27% of total singleton births over the study period) were included in analyses, with a mean (SD) maternal age of 28.8 (4.8) years. A total of 310 384 births (5.70%) were PTB, with a mean (SD) maternal age of 29.5 (5.5) years. Preterm birth was more common among mothers who lived in urban than rural areas (Table). Preterm birth was also more common in counties with a high GDP, high GDP per capita, high population, and low NDVI (eTable 3 in Supplement 1). Daily Tmax, Tmin, and relative humidity showed slight variation between rural and urban areas. Meanwhile, considerable variations were found among the 3 subnational socioeconomic regions (eFigure 2 in Supplement 1). eTable 2 in Supplement 1 shows that the numbers of PTB case and control days varied depending on the definition of heat waves.

**Table.** Demographic Characteristics of Preterm Birth in Rural and Urban Areas of China

Characteristic	Preterm births, No. (%)^a^		
	All	Rural	Urban
Total	310 384 (5.70)	73 756 (3.45)	236 628 (7.15)
Maternal age, y			
<25	54 613 (5.51)	21 058 (3.55)	33 555 (8.45)
25-34	197 573 (5.24)	42 200 (3.16)	155 373 (6.38)
≥35	58 198 (8.53)	10 498 (5.06)	47 700 (10.05)
Educational level			
Middle school or below	96 092 (5.43)	45 616 (3.58)	50 476 (10.21)
High school	87 635 (6.06)	19 352 (3.39)	68 283 (7.79)
College or higher	118 573 (5.60)	8636 (3.00)	109 937 (6.01)
Unknown	8084 (7.16)	152 (3.57)	7932 (7.30)
Subnational socioeconomic region			
High-income	110 720 (5.71)	26 873 (3.78)	83 847 (6.83)
Middle-income	97 397 (5.39)	24 749 (2.94)	72 648 (7.51)
Low-income	102 267 (6.02)	22 134 (3.78)	80 133 (7.20)

^a^ Percentages are calculated from numbers of all singleton births during the warm season (April to October) in China in each category for each demographic characteristic.

**Figure 2.**
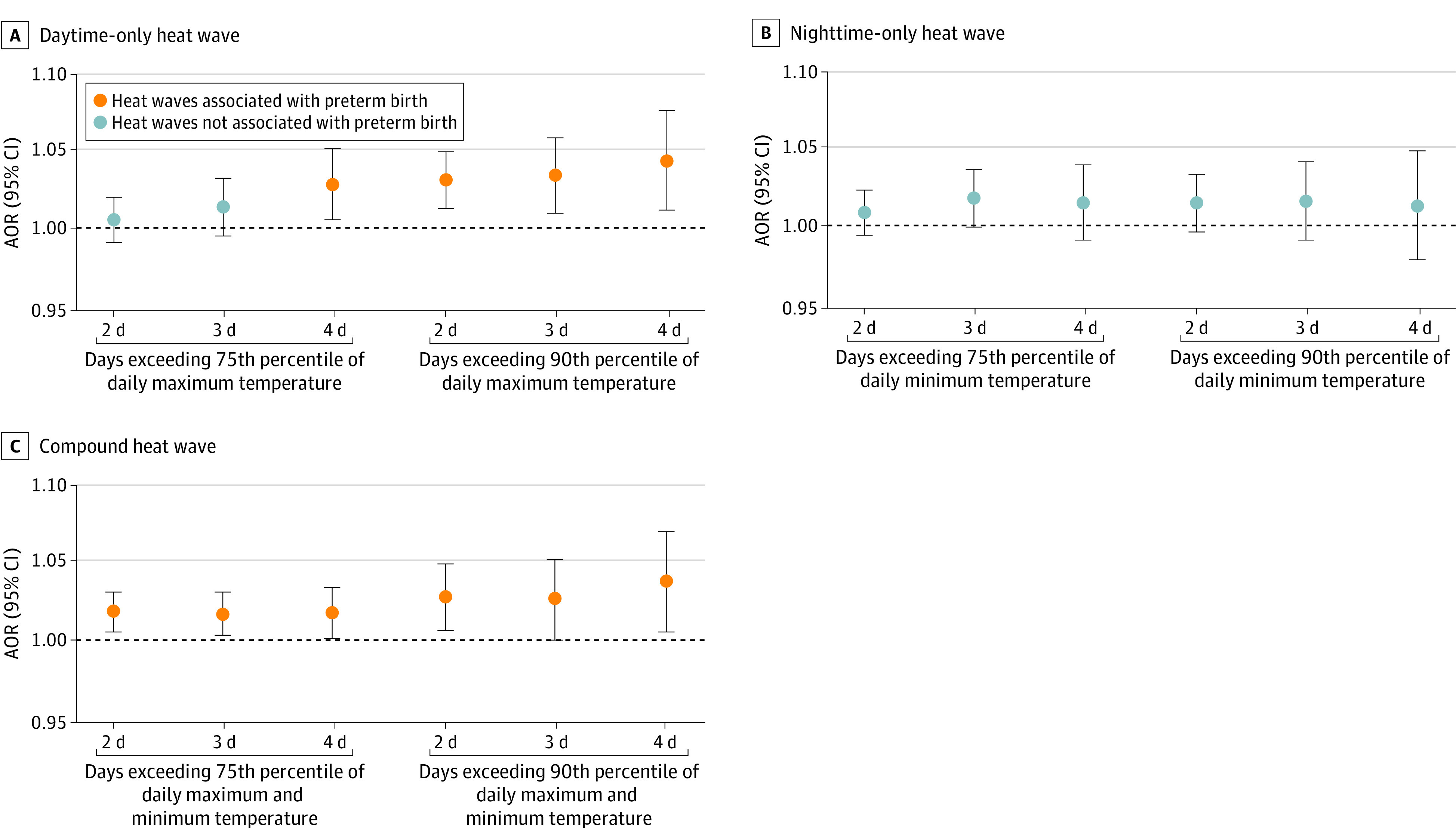
Adjusted Odds Ratios (AORs) of Preterm Birth Associated With 18 Definitions of Heat Waves During the Last Week Before Delivery A heat wave was defined as a period equal to or more than 2, 3, or 4 consecutive days above the daily temperature threshold. For daytime-only heat wave, days only exceed the 75th or 90th percentile of daily maximum temperature; nighttime-only heat wave, days only exceed the 75th or 90th percentile of daily minimum temperature; and compound heat wave, days exceed the 75th or 90th percentile of both daily maximum and minimum temperature. Models controlled the long-term trend, seasonality, effects of the day of the week, and time-invariant individual level confounders in design and adjusted for relative humidity and fine particulate matter with an aerodynamic diameter less than or equal to 2.5 μm using a natural cubic spline with 3 *df*. References for AORs were days of non–heat wave events under our definitions. We applied 1-stage analysis in multilocation. We estimated the overall AOR with the maximum points estimation in 7 lags (lag0, lag1, lag2, lag3, lag4, lag5, and lag6) under each heat wave definition. In subgroup analysis, we used the same lag as the estimation of overall as all subgroups, the maximum points estimation of AOR in 7 lags under each heat wave definition. Error bars indicate 95% CIs.

The adjusted OR (AOR) of PTB increased from 1.02 (95% CI, 1.00-1.03) to 1.04 (95% CI, 1.01-1.07) or following 6 indexes of compound heat waves during the last week before delivery (Figure 2). The association became steadily higher when thresholds rose and lasted longer. The AORs of PTB were only statistically significant following daytime-only heat waves with higher cutoff or longer duration. They increased from 1.03 (95% CI, 1.01-1.05) in 75th-D4 to 1.04 (95% CI, 1.01-1.08) in 90th-D4, which were slightly higher than in compound heat waves. We did not find associations between nighttime-only heat waves exposure and PTB. Results did not differ materially when we adjusted ozone and choices in *df* of the natural cubic spline for relative humidity or PM_2.5_ (eTable 5 in Supplement 1). eFigure 3 in Supplement 1 shows the AOR of preterm birth was elevated quickly and peaked in association with daytime-only heat waves 0 to 3 days preceding delivery. The association peaked in 3 to 6 days preceding delivery with exposure to compound heat waves (eTable 4 in Supplement 1).

**Figure 3.**
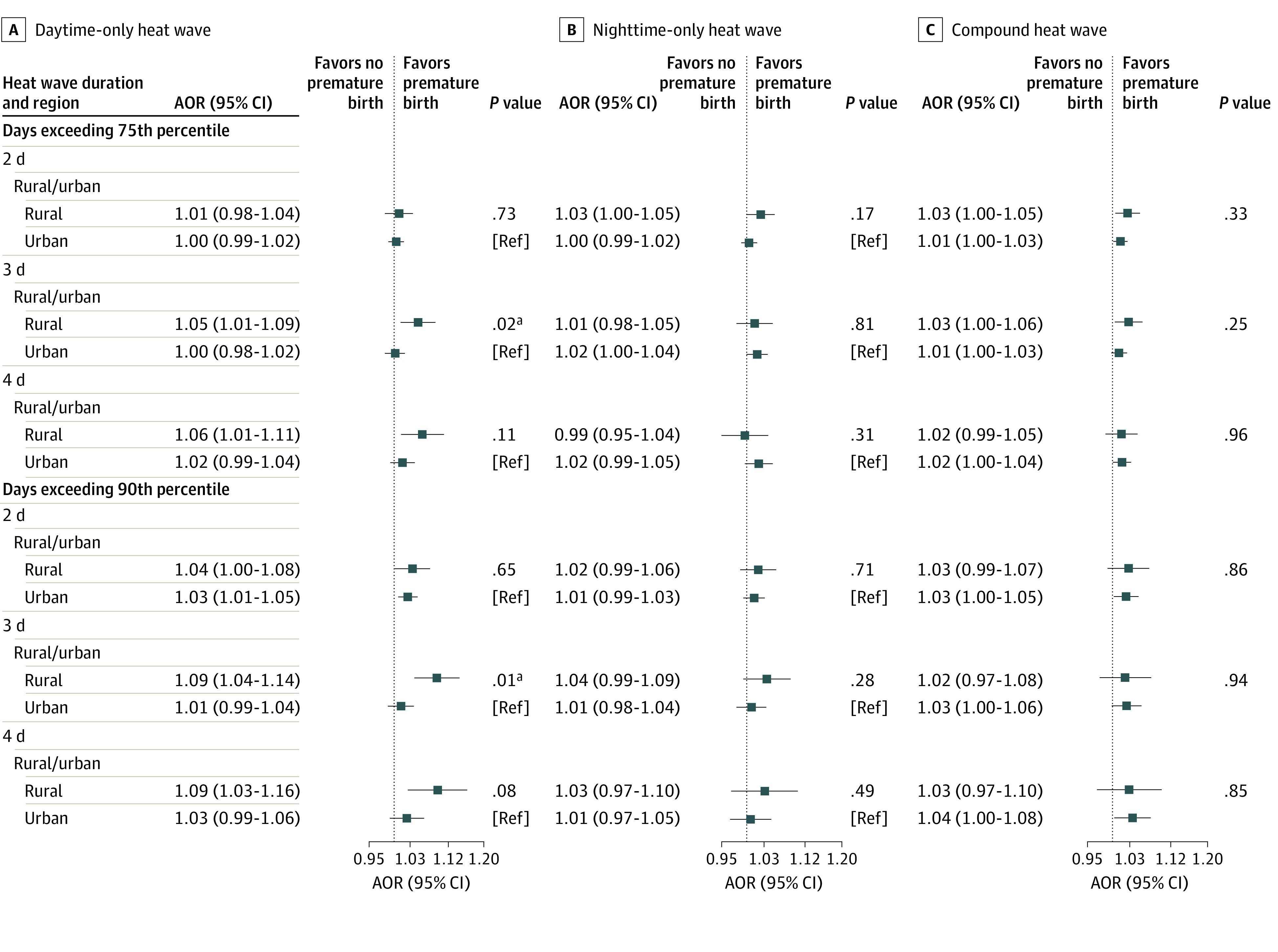
Adjusted Odds Ratios (AORs) of Preterm Birth Associated With Heat Waves During the Last Week Before Delivery in Urban and Rural Areas A heat wave was defined as a period equal to or more than 2, 3, or 4 consecutive days above the daily temperature threshold. For daytime-only heat wave, days only exceed the 75th or 90th percentile of daily maximum temperature; nighttime-only heat wave, days only exceed the 75th or 90th percentile of daily minimum temperature; and compound heat wave, days exceed the 75th or 90th percentile of both daily maximum and minimum temperature. Models controlled the long-term trend, seasonality, effects of the day of the week, and time-invariant individual level confounders in design and adjusted for relative humidity and fine particulate matter with an aerodynamic diameter less than or equal to 2.5 μm using a natural cubic spline with 3 *df*. References for AORs were days of non–heat wave events under our definitions. We applied 1-stage analysis in multilocation. We estimated the overall AOR with the maximum points estimation in 7 lags (lag0, lag1, lag2, lag3, lag4, lag5, and lag6) under each heat wave definition. In subgroup analysis, we used the same lag as the estimation of overall as all subgroups, the maximum points estimation of AOR in 7 lags under each heat wave definition. Ref indicates reference. ^a^Statistically significant.

**Figure 4.**
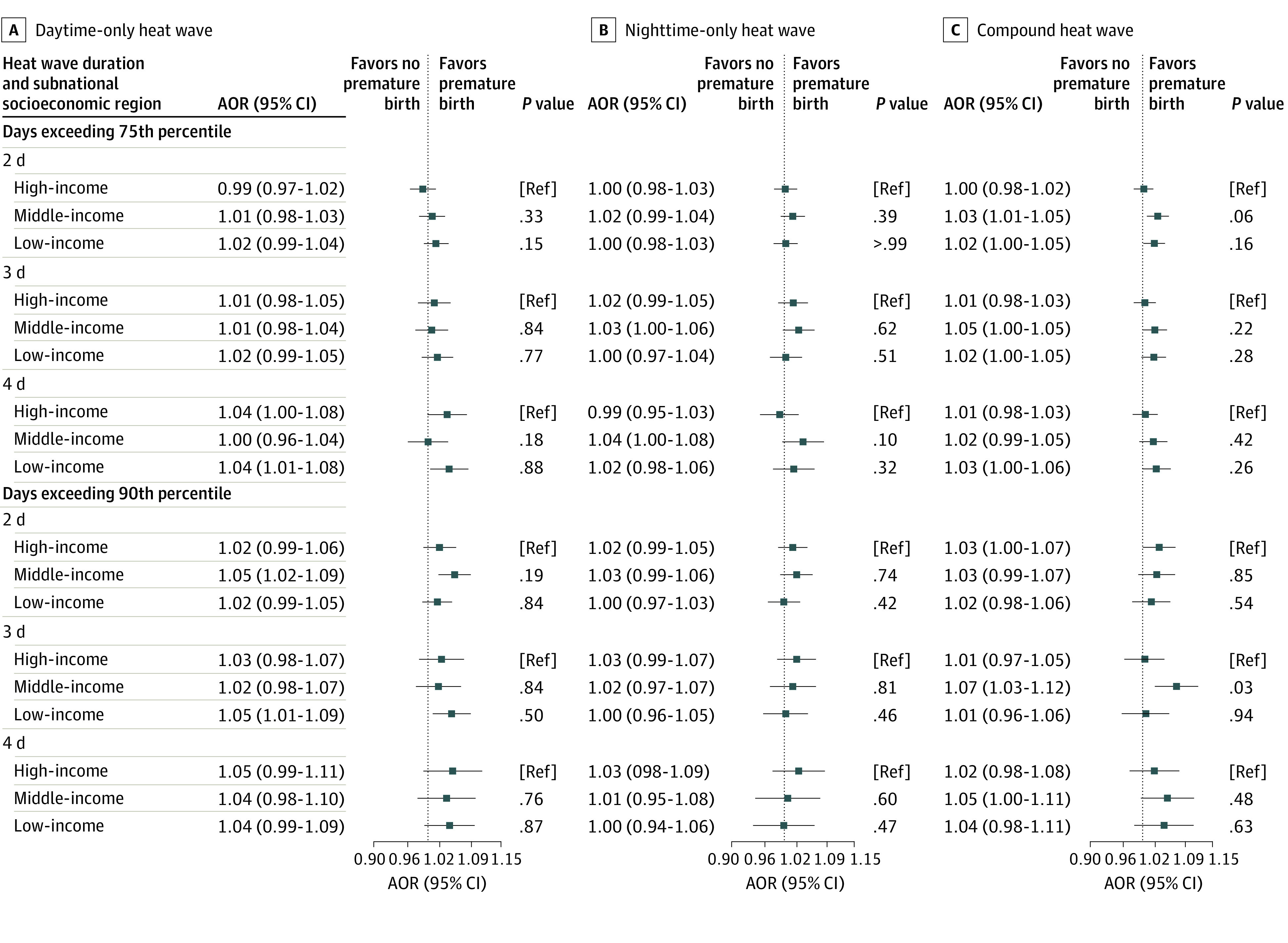
Adjusted Odds Ratios (AORs) of Preterm Birth Associated With Heat Waves During the Last Week Before Delivery Among Subnational Socioeconomic Regions A heat wave was defined as a period equal to or more than 2, 3, or 4 consecutive days above the daily temperature threshold. For daytime-only heat wave, days only exceed the 75th or 90th percentile of daily maximum temperature; nighttime-only heat wave, days only exceed the 75th or 90th percentile of daily minimum temperature; and compound heat wave, days exceed the 75th or 90th percentile of both daily maximum and minimum temperature. Models controlled the long-term trend, seasonality, effects of the day of the week, and time-invariant individual level confounders in design and adjusted for relative humidity and fine particulate matter with an aerodynamic diameter less than or equal to 2.5 μm using a natural cubic spline with 3 *df*. References for AORs were days of non–heat wave events under our definitions. We applied 1-stage analysis in multilocation. We estimated the overall AOR with the maximum points estimation in 7 lags (lag0, lag1, lag2, lag3, lag4, lag5, and lag6) under each heat wave definition. In subgroup analysis, we used the same lag as the estimation of overall as all subgroups, the maximum points estimation of AOR in 7 lags under each heat wave definition. Ref indicates reference.

Exposure to heat waves in rural areas was associated with a higher risk for PTB than in urban areas, except for compound heat waves in higher indexes (Figure 3 and eTable 6 in Supplement 1). The associations differ by urban-rural areas when exposed to daytime-only heat waves, with AORs of 1.05 (95% CI, 1.01-1.09) in rural areas compared with 1.00 (95% CI, 0.98-1.02) in urban areas in 75th-D3 and 1.09 (95% CI, 1.04-1.14) in rural areas compared with 1.01 (95% CI, 0.99-1.04) in urban areas in 90th-D3. When exposed to compound heat waves, the AORs of PTB ranged from 1.01 (95% CI, 1.00-1.03) to 1.04 (95% CI, 1.00-1.08) in 6 indexes for urban mothers, while the AORs for rural mothers ranged from 1.02 (95% CI, 0.99-1.05) to 1.03 (95% CI, 0.97-1.10), with no significant difference between urban and rural pregnant women. Results were similar in low GDP, low GDP per capita, low population, and high NDVI (eTable 6 in Supplement 1). We did not find statistically significant differences among socioeconomic regions in the associations of heat waves and PTB (Figure 4 and eTable 6 in Supplement 1).

## Discussion

In this national study of heat waves and PTB outcomes across diverse climate zones in China, we found that pregnant women were at a 1.6% (95% CI, 0.3%-3.0%) to 3.7% (95% CI, 0.5%-6.9%) higher risk for PTB when exposed to compound heat waves during the last week before delivery and a 2.7% (95% CI, 0.5%-5.0%) to 4.2% (95% CI, 1.1%-7.5%) higher risk when exposed to daytime-only heat waves, compared with those who were unexposed. Our findings in China are consistent with and expand on the results of previous studies.^1,2,3,4,5,8,16^ A recent meta-analysis^8^ found 1.16-fold higher odds of PTB during heat waves. Seven of 8 prior single city or state studies in the US, Australia, Italy, China, and Iran that evaluated the association between heat waves and PTB^1,9,37,46,47,48,49^ found a 2.1% (95% CI, 0.8%-3.5%) to 92% (95% CI, 39%-164%) higher risk for PTB compared with non–heat wave days. In contrast, another Canadian study^50^ found a null association. Moreover, a multisite study in 50 US metropolitan areas^2^ found no association with the limitation of using 1980s data. Prior studies^1,2,9,37^ have found increasing associations for various definitions of heat waves while the thresholds and duration increased, consistent with our findings. For heat waves that exceeded the 75th percentile and lasted at least 2 days, 1 prior study in Guangzhou, China,^1^ found a 15% (95% CI, 5%-25%) higher risk. In contrast, another study in California^37^ found a null association. Six prior studies^1,9,37,46,48,49^ found a 2.2% (95% CI, 0.7%-3.7%) to 37% (95% CI, 20%-55%) higher risk for heat waves defined with 90th percentile and at least 2 days, while 1 prior study in Alabama^9^ found a null association.

Previous studies^2,8^ have not uncovered a risk of PTB after prenatal exposure to daytime heat combined with nighttime heat or lack thereof. Moreover, an analysis of the association between PTB and hourly increased temperature^51^ observed a higher risk of PTB with a temperature increase during early morning in the warm season. We split traditionally defined heat waves into 3 exclusive types to unmask this issue: daytime-only, nighttime-only, or compound heat waves. We found consistently positive associations between compound heat waves and the risk of PTB in China. Meanwhile, exposure to daytime-only heat waves was only associated with PTB at a higher cutoff or a longer duration. The risk of PTB was slightly larger than exposure to compound heat waves. Such findings will provide needed scientific evidence for clinical practice development to protect maternal health and prevent PTB.

Our study also addresses knowledge gaps in the literature about the urban-rural and regional socioeconomic disparities analysis regarding the association between heat exposure and PTB. Most previous studies investigated urban populations,^8^ with only a few focusing on rural populations.^5,6,9,14,52^ Two studies in China based on the same project, the National Free Prepregnancy Checkups Project, which enrolled 94% of participants from rural areas,^5,52^ found a 1.9% (95% CI, −6.7% to 11.3%) to 6.9% (95% CI, 1.0%-13.2%) higher risk of PTB associated with high temperatures compared with moderate temperatures. Updating that, our study found that compared with pregnant women in rural areas, those in urban areas had a lower risk of PTB after exposure to heat waves, except for compound heat waves in higher indexes. Though effect modification by rurality for heat or heat wave–related PTB is largely inconsistent,^6,9,14,15^ our study found statistically significant urban-rural differences in the risk of PTB after daytime-only heat wave exposure.

Our findings of a higher risk of PTB after exposure to heat waves in the counties or districts with lower SES are broadly consistent with the previous studies,^13,14,15,16^ which will have significant clinical and maternal health implications. Four prior heat-PTB studies reported similar findings in North Carolina^14^; Rome, Italy^13^; Seoul, Korea^15^; and 8 cities in China^16^ and found a 1.8% (95% CI, 0.6%-2.9%) to 4.9% (95% CI, 0.9%-8.9%) higher risk in areas with low SES levels compared with a 0.4% (95% CI, −1.7% to 0.8%) to 1.6% (95% CI, −2.2% to 5.5%) higher risk in areas with high SES levels. These studies have mixed settings on definitions of heat and heat wave, exposure window, SES indicators, and the area’s magnitude in area-level analysis. We also found that the association between heat waves and PTB risk was markedly larger in lower county-level SES but unvaried in regional-level SES.

The physiological heat stress responses include dehydration, fluid loss, and cardiovascular strain.^53,54^ A decreased body surface area to mass ratio during pregnancy may be related to the smaller capacity for evaporative cooling in heat stress.^4,54^ Cardiovascular changes in pregnancy include increased heart rate and higher blood pressure, especially in the third trimester.^55^ Decreased thermoregulatory ability and increased cardiovascular changes may make pregnant women more vulnerable to heat stress. Nevertheless, how acute prenatal exposure affects preterm birth is still unclear.^4^ The heat-caused dehydration during pregnancy may decrease uterine oxygen and induce uterine contractions to labor.^4,55^ Meanwhile, consecutive days with high overnight temperature risk increase vulnerability to heat stress at lower temperatures because higher nighttime temperatures preclude the heat stress relief provided by cool night temperatures.^53^ That may contribute to associations between compound heat waves and PTB. A higher risk for PTB in compound heat waves in urban areas, only in higher indexes, may also be owing to amplification of the urban heat island at night when stored daytime heat is emitted and intensifies with increasing city size and population density.^54^ Potential sources of urban-rural disparities may partly contribute to factors correlated with urbanization levels, such as available medical resources, working places’ cooling conditions, and households’ rates of air conditioner use.^56,57,58^ Moreover, behavioral factors may also be related to these inequalities. Rural pregnant women may also endure the uncertainty of moving to cool public areas that may provide physiological respite.^54^

### Strengths and Limitations

Our study has several strengths. First, we analyzed disparities of associations between heat waves and PTB based on a representative national sampling database in the largest developing country in the world with data on more than 10 million births, and the results provide compelling evidence for the research field. Second, we considered human climate adaptation by setting the reference period as 1981 to 2010, the recent climate state period and meteorological variation, by setting a 15-day window (7 days prior and 7 days later to a specific day) to get 450 samples in defining daily extremes thresholds. Third, we categorized distinct types of heat waves to uncover trends masked in traditionally defined heat waves by daily Tmax or Tmin.

The limitations of this study should be acknowledged. First, only the addresses of health facilities at the time of delivery instead of the mothers’ residences were available in the NMNMSS, which may lead to potential misclassification bias for previous days. To minimize such exposure misclassification, we calculated mean grid cell values from the 25-km radius around each health facility’s address to assign individual exposure and cover possible pregnancy residences in the preceding days. Maternal case notes from rural and urban areas showed that a shorter distance from the residence was a substantial factor when pregnant women chose health facilities for delivery. About 80% of pregnant women gave birth in health facilities in their home city.^35,59^ Additionally, varied spatial resolution, postal codes, and 12.5 and 1 km of the exposure estimation may produce similar association estimates between heat waves and preterm birth.^60^ Second, the case-crossover design only estimates a short-term association of heat with PTB because this design is suited to the association between intermittent exposures with short induction times and transient acute effects.^40^ Long-term risks in the same study population will be estimated elsewhere. Third, our study is exploratory to examine associations between exposure to 18 definitions of heat waves in the last week before delivery and PTB and lag effects. The AORs for our findings were significant but relatively small. Further research to confirm these findings is needed.

## Conclusions

The findings of this case-crossover study of pregnant women in China suggest that exposure to compound and daytime-only heat waves in the last week before delivery is associated with PTB, especially for women who live in rural areas. These results add to the growing evidence identifying extreme heat as an essential factor associated with PTB in the context of climate change and may also be useful for urban-rural prevention strategies, protecting pregnant women from heat waves to decrease heat-related PTB in China.

## Notes

Supplement 1. **eMethods.** Population and Definitions **eFigure 1.** Flow Diagram of Birth Exclusion **eFigure 2.** Daily Ambient Maximum Temperature, Minimum Temperature, and Mean Relative Humidity in the Warm Season (April to October) During 2013 to 2019 **eFigure 3.** Lag Day-Specific Associations Between Heat Waves and Preterm Births **eTable 1.** Number of Sampled Health Facilities and Counties or Districts in Rural, Urban, and Subnational Socioeconomic Regions **eTable 2.** Number of Case and Control Days of Preterm Birth After Exposure to Heat Waves in the Warm Season (April to October) in 438 Sampled Sites of China During 2012 to 2019 **eTable 3.** Characteristics of Preterm Birth in Counties or Districts of Low and High GDP, GDP per Capita, Population, and NDVI **eTable 4.** Lag Day With the Maximum Points Estimation of the Overall Odds Ratios in 19 Definitions of Heat Waves **eTable 5.** Sensitivity Analysis by Testing Different Modeling Choices **eTable 6.** Adjusted Odds Ratios of Preterm Birth Associated With Heat Waves and *P* Values for Effect Modification by Urban, Rural, and Subnational Socioeconomic Regions
eReferences

Supplement 2.
Data Sharing Statement
