# Supplementary material for: Association of Daytime-Only, Nighttime-Only, and Compound Heat Waves With Preterm Birth by Urban-Rural Area and Regional Socioeconomic Status in China
Source: JAMA Netw Open. 2023 Aug 11;6(8):e2326987. doi: 10.1001/jamanetworkopen.2023.26987 (PMC10422195; doi:10.1001/jamanetworkopen.2023.26987)
Supplement: Supplement 1. — eMethods. Population and Definitions eFigure 1. Flow Diagram of Birth Exclusion eFigure 2. Daily Ambient Maximum Temperature, Minimum Temperature, and Mean Relative Humidity in the Warm Season (April to October) During 2013 to 2019 eFigure 3. Lag Day-Specific Associations Between Heat Waves and Preterm Births eTable 1. Number of Sampled Health Facilities and Counties or Districts in Rural, Urban, and Subnational Socioeconomic Regions eTable 2. Number of Case and Control Days of Preterm Birth After Exposure to Heat Waves in the Warm Season (April to October) in 438 Sampled Sites of China During 2012 to 2019 eTable 3. Characteristics of Preterm Birth in Counties or Districts of Low and High GDP, GDP per Capita, Population, and NDVI eTable 4. Lag Day With the Maximum Points Estimation of the Overall Odds Ratios in 19 Definitions of Heat Waves eTable 5. Sensitivity Analysis by Testing Different Modeling Choices eTable 6. Adjusted Odds Ratios of Preterm Birth Associated With Heat Waves and P Values for Effect Modification by Urban, Rural, and Subnational Socioeconomic Regions eReferences [file jamanetwopen-e2326987-s001.pdf]

Supplementary Online Content

Guo Y, Chen P, Xie Y, et al. Association of daytime-only, nighttime-only, and compound heat waves with preterm birth by urban-rural area and regional socioeconomic status in China. *JAMA Netw Open*. 2023;6(8):e2326987. doi:10.1001/jamanetworkopen.2023.26987

eMethods. Population and Definitions

eFigure 1. Flow Diagram of Birth Exclusion

eFigure 2. Daily Ambient Maximum Temperature, Minimum Temperature, and Mean Relative Humidity in the Warm Season (April to October) During 2013 to 2019

eFigure 3. Lag Day-Specific Associations Between Heat Waves and Preterm Births

eTable 1. Number of Sampled Health Facilities and Counties or Districts in Rural, Urban, and Subnational Socioeconomic Regions

eTable 2. Number of Case and Control Days of Preterm Birth After Exposure to Heat Waves in the Warm Season (April to October) in 438 Sampled Sites of China During 2012 to 2019

eTable 3. Characteristics of Preterm Birth in Counties or Districts of Low and High GDP, GDP per Capita, Population, and NDVI

eTable 4. Lag Day With the Maximum Points Estimation of the Overall Odds Ratios in 19 Definitions of Heat Waves

eTable 5. Sensitivity Analysis by Testing Different Modeling Choices

eTable 6. Adjusted Odds Ratios of Preterm Birth Associated With Heat Waves and *P* Values for Effect Modification by Urban, Rural, and Subnational Socioeconomic Regions

eReferences

This supplementary material has been provided by the authors to give readers additional information about their work.

© 2023 Guo Y et al. *JAMA Network Open*.

eMethods. Population and Definitions

Sampling of China's National Maternal Near Miss Surveillance System (NMNMSS)

China's National Maternal Near Miss Surveillance System (NMNMSS) was a health surveillance system initiated in 2010 to collect each admitted pregnant woman's health information with the form filled by the clinician. The NMNMSS used three steps to recruit participants: randomly selected rural counties or urban districts within three subnational regions, then randomly selected one or two health facilities with more than 1000 births per year within each sampled county or district, and finally, recruited all pregnant women admitted to the obstetric department of sampled health facilities and collected individual survey forms filled out by attending clinician. Participants were followed up until they were discharged from the hospital. According to China's National Census 2010, it covered about 13% of the population in 30 provinces of mainland China except for Tibet.[1-3]

Urban-rural classification

Urban-rural classification depended on three administrative types of cities: provincial-level (consisting of municipalities and special administrative regions), prefecture-level, and county-level cities. In our study, we followed China's National Health Statistical Yearbook,[4] the urban area included districts of provincial- and prefecture-level cities, and the rural area included counties and county-level cities.

Provincial-level is the top administrative level, followed by prefecture- and county-level.[5] Provincial-level cities are centrally administered cities with the same administrative status as provinces. There are four centrally administered cities in mainland China: Beijing, Shanghai, Tianjin, and Chongqing. A prefecture-level city is a large urban area that serves as the administrative center for a group of smaller county-level cities, counties, and urban districts. There are around 300 prefecture-level cities in China. Only urban districts of prefecture-level cities are classified as urban areas in our study. Counties and all county-level cities are classified as rural areas.

In addition to the administrative measure, we also calculated county-level annual gross domestic product (GDP), GDP per capita, population, and warm season normalized difference vegetation index (NDVI) to measure rurality. We derived yearly GDP and population and monthly NDVI from gridded datasets at 1km resolution and calculated GDP per capita gridded data equal to GDP divided by population.[6-8] China's GDP and population gridded datasets were spatialized from national GDP and population statistical data and weighted by night light brightness and residential density.[6,7] China's NDVI monthly gridded datasets were generated from 1 km 10–day synthesis data products of Proba-V (Project for On-Board Autonomy Vegetation).[8] To calculate the county-level value, we took an average of 1km grid cells within a district or county. County-level GDP, GDP per capita, and population were estimated for 2015, the middle year of our study period. And we counted the warm season NDVI for April to October 2015. We use the median GDP, GDP per capita, population, and NDVI to divide high and low for analysis.

Subnational regions classification

According to China's National Health Statistical Yearbook,[4] the eastern region includes Beijing, Tianjin, Hebei, Liaoning, Shanghai, Jiangsu, Zhejiang, Fujian, Shandong, Guangdong, and Hainan; the central region includes Shanxi, Jilin, Heilongjiang Anhui, Jiangxi, Henan, Hubei, and Hunan; and the western region includes Inner Mongolia, Chongqing, Guangxi, Sichuan, Guizhou, Yunnan, Tibet, Shaanxi, Gansu, Qinghai, Ningxia and Xinjiang. Eastern, Central, and Western regions represented high-, middle- and low-income regions in our study.

© 2023 Guo Y et al. *JAMA Network Open*.

Definition of three types and six indices of heatwaves

We followed three steps to calculate the daily binary variables to indicate whether it is a heatwave day of 18 definitions (three types in six indices) of heatwaves, within each health facility's 25 km radius domain during the study period 2012–2019. Firstly, we calculated daily Tmax (daily maximum temperature) and Tmin (daily minimum temperature) in each domain during 1981–2010 and 2012–2019. Secondly, we estimated daily thresholds, 75th or 90th percentile of daily maximum temperature or daily minimum temperature, within each health facility's 25 km radius domain, by taking 1981–2010 as the reference period. Thirdly, we appointed daily indicators of 18 definitions of heatwaves for each domain during the study period 2012–2019.

Daily thresholds were calculated base on the reference period, 1981–2010. For a given day, we took a window of seven days before and seven days after for 15 temperature values associated with each day in each year of the reference period, 1981–2010. So, we generated a sample set of 15 days multiply 30 years, equal to 450 temperature values as the reference windows for each day. We calculated percentiles for each of these sets of 450 values to define daily thresholds.

$$TX_d = \bigcup_{y=1981}^{2010} \bigcup_{i=d-7}^{d+7} Tmax_{y,i}$$

$$TN_d = \bigcup_{y=1981}^{2010} \bigcup_{i=d-7}^{d+7} Tmin_{y,i}$$

For a given day d in a grid cell, the threshold, TX_d, and TN_d are defined as the 75th, or 90th percentile of daily maximum or minimum temperature, centered on a 15-day window (seven days prior and seven days later to a specific day) in the reference period 1981–2010, which has 15 multiply 30 equals to 450 samples.

where $\bigcup$ denotes the union of 450 sample sets for the given day d;

$Tmax_{y,i}$ is the daily Tmax of the day i in the year y;

$Tmin_{y,i}$ is the daily Tmin of the day i in the year y.

© 2023 Guo Y et al. *JAMA Network Open*.

eFigure 1. Flow Diagram of Birth Exclusion

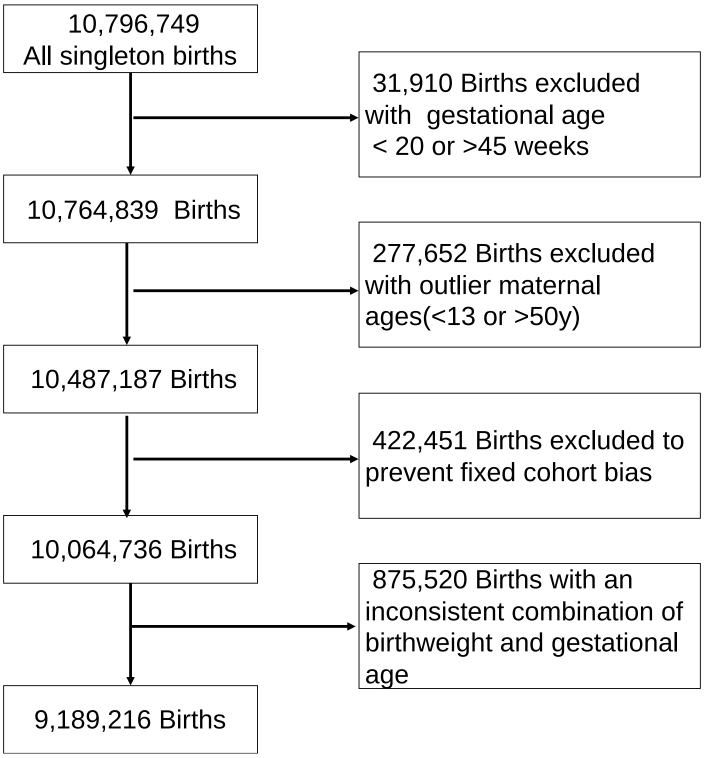

We used four exclusion criteria, births with gestational age < 20 or >45 weeks (N = 31,910, 0.3%); births with maternal ages (<13 or >50y) depending on the average age at menarche (12.8y) and the median age at natural menopause (50y) of women (N = 277,652, 2.6%);[9,10] births with conception dates earlier than 20 weeks prior to Jan 1, 2012 and later than 45 weeks before Dec 31, 2019 to limit potential fixed cohort bias (N = 422,451, 4.0%);[11] births with an inconsistent combination of birthweight and gestational age according to growth standard curves of birth weight of Chinese newborns of different gestation (N = 875,520, 8.7%)[12].

© 2023 Guo Y et al. *JAMA Network Open*.

eFigure 2. Daily Ambient Maximum Temperature, Minimum Temperature, and Mean Relative Humidity in the Warm Season (April to October) During 2013 to 2019

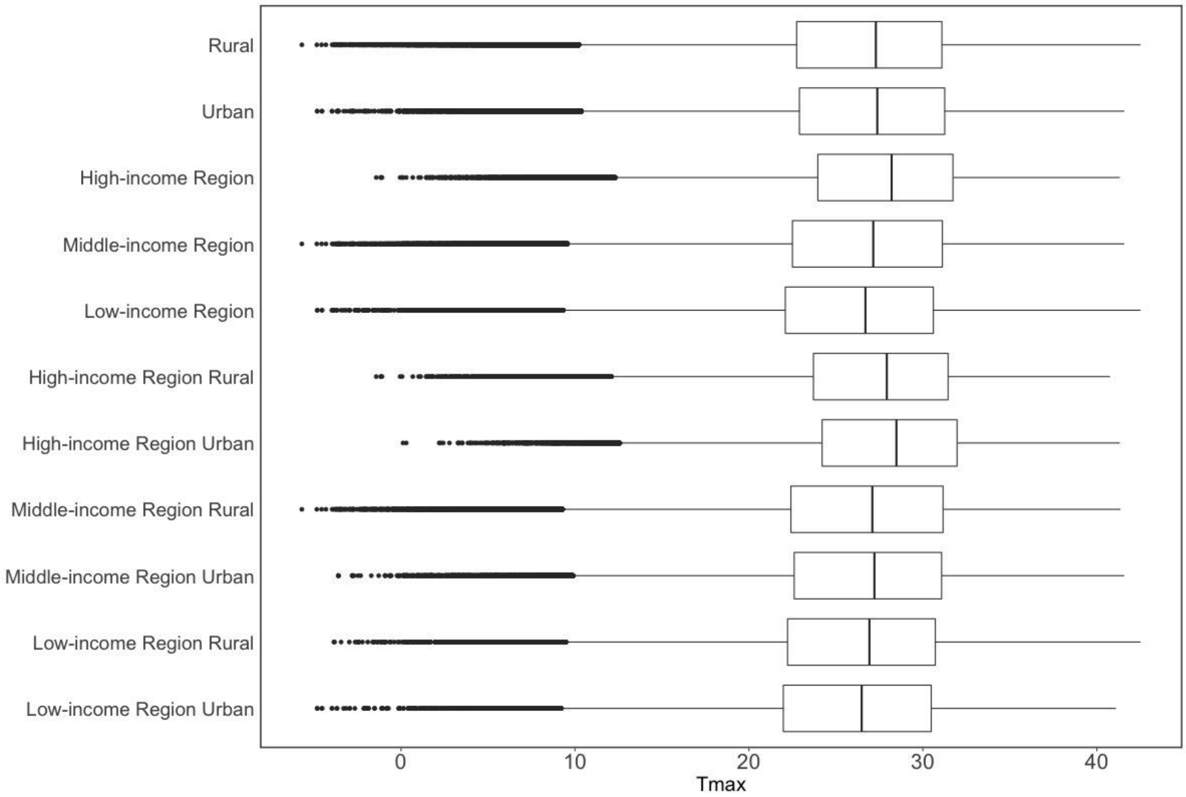

Daily maximum temperature (℃)

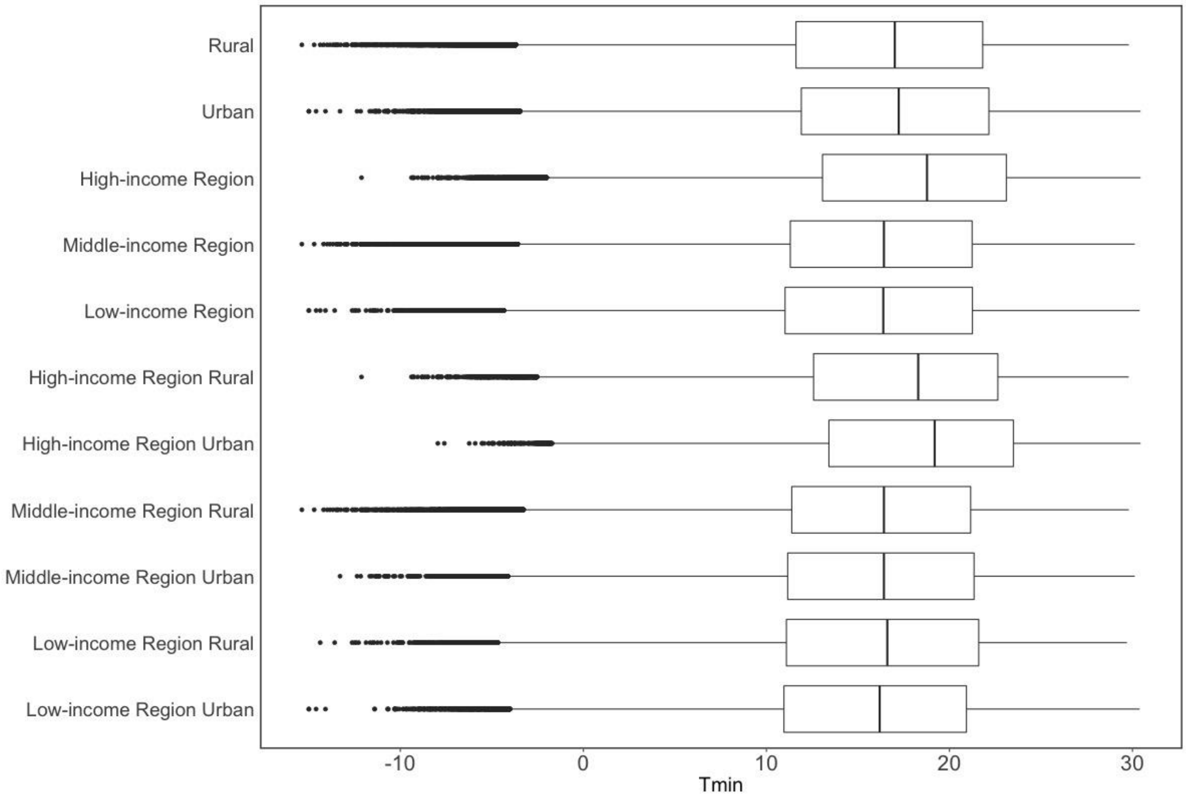

Daily minimum temperature (℃)

© 2023 Guo Y et al. *JAMA Network Open*.

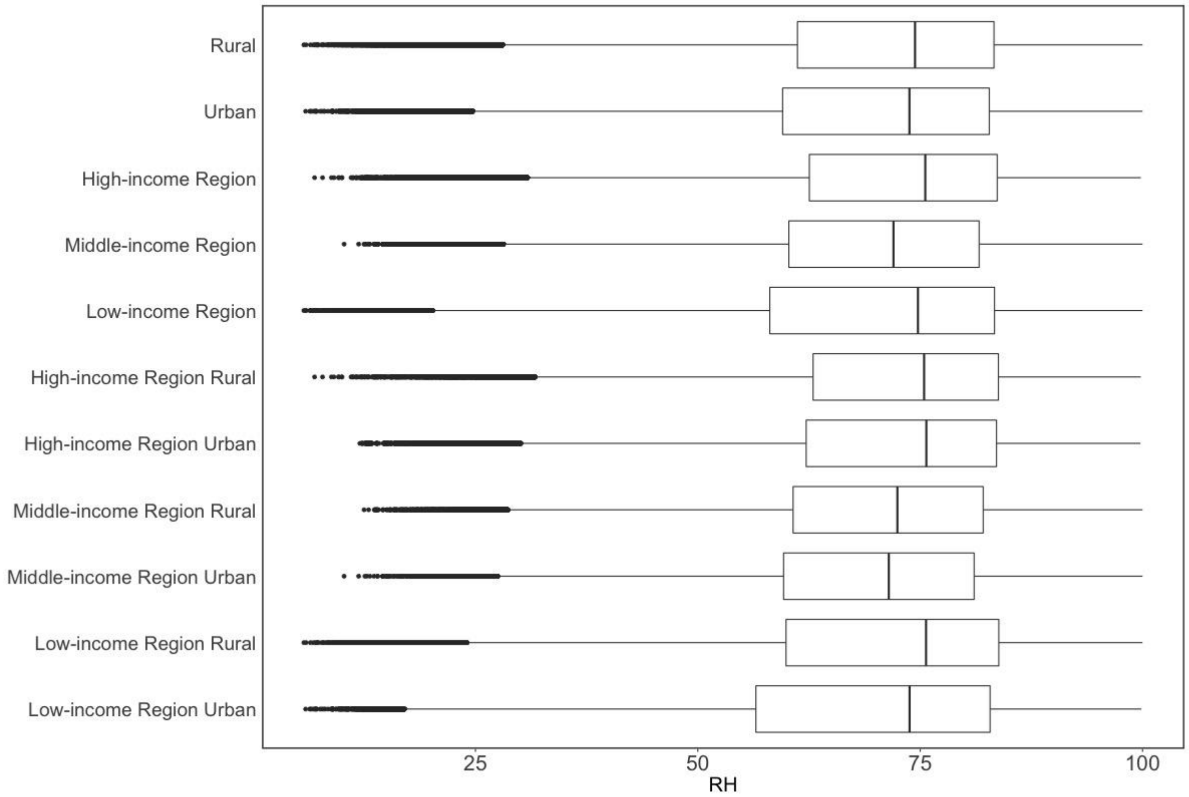

Daily mean relative humidity (%)

Boxes of the box plots cover the interquartile ranges with a center line for the median. Whiskers of the box plots extend to the minimum and maximum observations, with outliers shown as dots. The daily maximum temperature of rural plotted rural sampled sites' daily maximum temperature from April 1st to October 30th during 2012–2019. Others are similar.

© 2023 Guo Y et al. *JAMA Network Open*.

eFigure 3. Lag Day-Specific Associations Between Heat Waves and Preterm Births

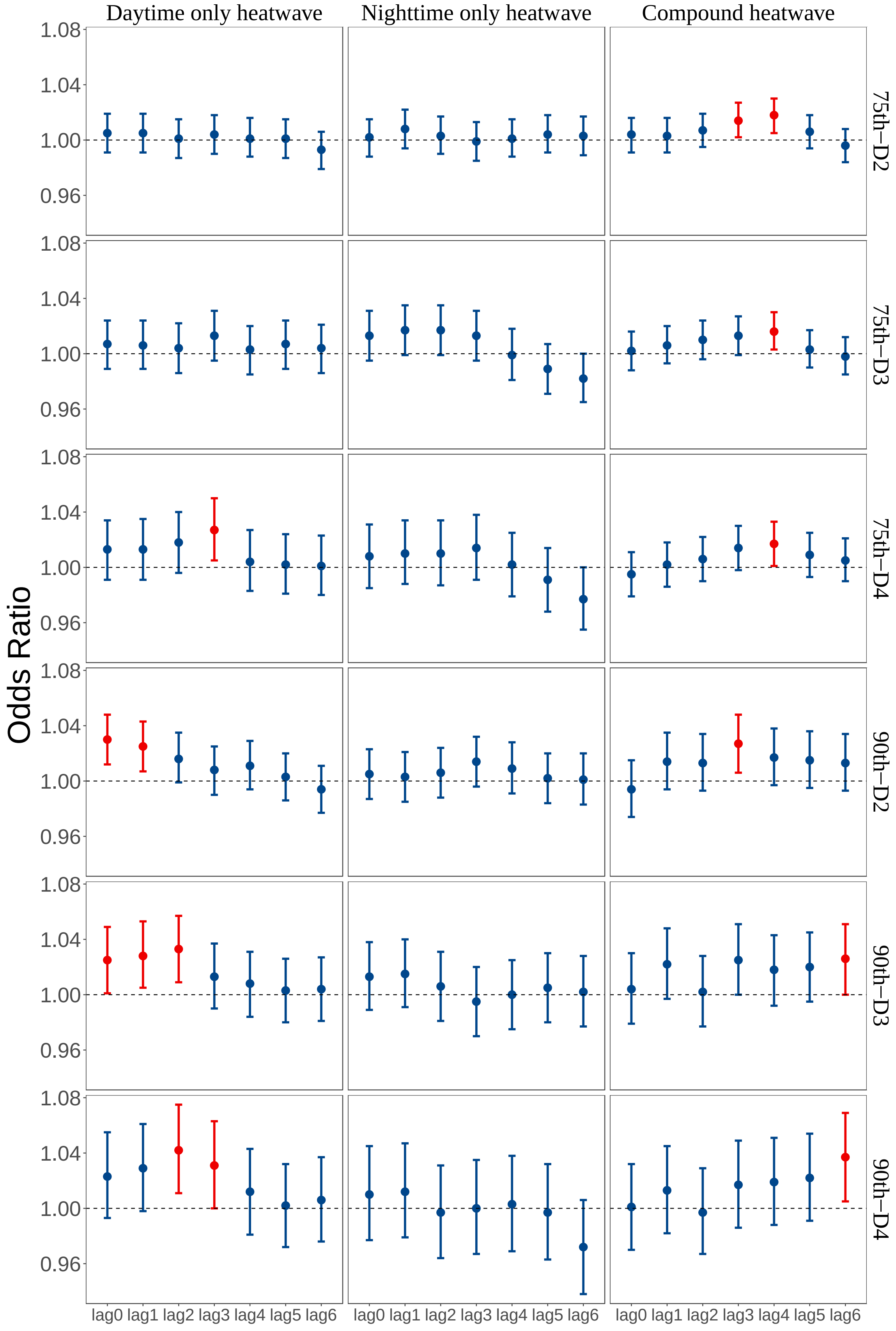

© 2023 Guo Y et al. *JAMA Network Open*.

Red color indicated positive associations between heat waves and preterm birth. Blue color indicated non-significant associations between heat waves and preterm birth. A heat wave was defined as a period equal to or more than two, three, or four consecutive days above the daily threshold. Daytime-only heatwave contains only the 75th or 90th percentile of daily maximum temperature. Nighttime-only heatwave has only the 75th or 90th percentile of daily minimum temperature. Compound heatwave contains 75th or 90th percentile of daily maximum and minimum temperature. The 75th-D2 daytime-only heatwave is a period that only has equal to or more than two consecutive days above the 75th percentile of daily maximum temperature. Others are defined similarly. Models controlled the long-term trend, seasonality, effects of the day of the week, and time-invariant individual level confounders in design and adjusted for relative humidity and $PM_{2.5}$ using a natural cubic spline with 3 degrees of freedom.

© 2023 Guo Y et al. *JAMA Network Open*.

eTable 1. Number of Sampled Health Facilities and Counties or Districts in Rural, Urban, and Subnational Socioeconomic Regions

	Number of sampled health facilities	Number of sampled counties or districts
**Overall**	438	325
**Rural/Urban**		
Rural	223	170
Urban	215	155
**Subnational Socioeconomic Regions**		
High-income Region	142	104
Middle-income Region	140	108
Low-income Region	156	113
**GDP per capita**		
Low	208	163
High	230	162
**GDP**		
Low	208	162
High	230	163
**Population**		
Low	209	163
High	229	162
**NDVI**		
Low	221	162
High	217	163

© 2023 Guo Y et al. *JAMA Network Open*.

eTable 2. Number of Case and Control Days of Preterm Birth After Exposure to Heat Waves in the Warm Season (April to October) in 438 Sampled Sites of China During 2012 to 2019

Heatwave definitions[a]		Case days(n)[b]	Control days(n)[b]
**Indices**	**Types**		
75th-D2	Daytime-only heatwave	33,499	114,621
	Nighttime-only heatwave	32,330	110,247
	Compound heatwave	43,548	148,245
75th-D3	Daytime-only heatwave	20,819	71,146
	Nighttime-only heatwave	17,867	60,445
	Compound heatwave	33,407	113,864
75th-D4	Daytime-only heatwave	13,414	45,431
	Nighttime-only heatwave	10,150	34,200
	Compound heatwave	24,469	83,306
90th-D2	Daytime-only heatwave	19,256	64,265
	Nighttime-only heatwave	17,015	57,446
	Compound heatwave	13,230	44,659
90th-D3	Daytime-only heatwave	10,443	34,849
	Nighttime-only heatwave	8,856	30,009
	Compound heatwave	8,907	30,121
90th-D4	Daytime-only heatwave	6,035	19,962
	Nighttime-only heatwave	4,662	15,787
	Compound heatwave	5,807	19,410

[a]A heat wave was defined as a period equal to or more than two, three, or four consecutive days above the daily threshold. Daytime-only heatwave contains only the 75th or 90th percentile of daily maximum temperature. Nighttime-only heatwave has only the 75th or 90th percentile of daily minimum temperature. Compound heatwave contains 75th or 90th percentile of daily maximum and minimum temperature. The 75th-D2 daytime-only heatwave is a period that only has equal to or more than two consecutive days above the 75th percentile of daily maximum temperature. Others are defined similarly. [b]Number of case days and control days counted case and control days under the specific heat wave definition in all sampled sites. The delivery date of preterm birth was case day. Control days comprised the same day of the week in the calendar month of preterm birth onset. Then each case day would have three or four control days.

© 2023 Guo Y et al. *JAMA Network Open*.

eTable 3. Characteristics of Preterm Birth in Counties or Districts of Low and High GDP, GDP per Capita, Population, and NDVI

	Preterm births, n (%)
**GDP per capita, thousand RMB per person in 2015**	
Low (< 45)	85,235 (4.16)
High (>= 45)	225,149 (6.62)
**GDP, thousand RMB per square kilometer in 2015**	
Low (< 20,738)	64,703 (3.62)
High (>= 20,738)	245,681 (6.72)
**Population, persons per square kilometer in 2015**	
Low (< 486)	65,332 (3.70)
High (>= 486)	245,052 (6.64)
**NDVI, index in warm season of 2015**	
Low (< 0.56)	206,468 (6.63)
High (>= 0.56)	103,916 (4.46)

© 2023 Guo Y et al. *JAMA Network Open*.

eTable 4. Lag Day With the Maximum Points Estimation of the Overall Odds Ratios in 18 Definitions of Heat Waves

Heatwave definitions[a]		Lag day[b]
**Indices**	**Types**	
75th-D2	Daytime-only heatwave	lag1
	Nighttime-only heatwave	lag1
	Compound heatwave	lag4
75th-D3	Daytime-only heatwave	lag3
	Nighttime-only heatwave	lag2
	Compound heatwave	lag4
75th-D4	Daytime-only heatwave	lag3
	Nighttime-only heatwave	lag3
	Compound heatwave	lag4
90th-D2	Daytime-only heatwave	lag0
	Nighttime-only heatwave	lag3
	Compound heatwave	lag3
90th-D3	Daytime-only heatwave	lag2
	Nighttime-only heatwave	lag1
	Compound heatwave	lag6
90th-D4	Daytime-only heatwave	lag2
	Nighttime-only heatwave	lag1
	Compound heatwave	lag6

[a]A heat wave was defined as a period equal to or more than two, three, or four consecutive days above the daily threshold. Daytime-only heatwave contains only the 75th or 90th percentile of daily maximum temperature. Nighttime-only heatwave has only the 75th or 90th percentile of daily minimum temperature. Compound heatwave contains 75th or 90th percentile of daily maximum and minimum temperature. The 75th-D2 daytime-only heatwave is a period that only has equal to or more than two consecutive days above the 75th percentile of daily maximum temperature. Others are defined similarly. [b]Lag0 was the delivery day, lag1 was the day before delivery, others are similar, and lag6 was the sixth day before delivery.

© 2023 Guo Y et al. *JAMA Network Open*.

eTable 5. Sensitivity Analysis by esting Different Modeling Choices

Heatwave definitions[a]		Main model[b]	Main model+O_3[c]	DF/humidity: 3<br>DF/ $PM_{2.5}$: 4	DF/humidity: 3<br>DF/ $PM_{2.5}$: 5	DF/humidity: 4<br>DF/ $PM_{2.5}$: 5
Indices	Types					
75th-D2	Daytime-only heatwave	1.005 (0.991, 1.019)	1.011 (0.996, 1.025)	1.005 (0.991, 1.019)	1.005 (0.991, 1.019)	1.005 (0.991, 1.019)
	Nighttime-only heatwave	1.008 (0.994, 1.022)	1.009 (0.994, 1.024)	1.008 (0.994, 1.022)	1.008 (0.994, 1.022)	1.008 (0.994, 1.022)
	Compound heatwave	1.018 (1.005, 1.030)	1.017 (1.004, 1.030)	1.018 (1.005, 1.030)	1.018 (1.005, 1.030)	1.018 (1.006, 1.030)
75th-D3	Daytime-only heatwave	1.013 (0.995, 1.031)	1.013 (0.994, 1.031)	1.013 (0.995, 1.031)	1.013 (0.995, 1.031)	1.013 (0.995, 1.031)
	Nighttime-only heatwave	1.017 (0.999, 1.035)	1.013 (0.994, 1.033)	1.017 (0.999, 1.035)	1.017 (0.999, 1.035)	1.017 (0.999, 1.035)
	Compound heatwave	1.016 (1.003, 1.030)	1.014 (1.000, 1.029)	1.016 (1.003, 1.030)	1.016 (1.003, 1.030)	1.016 (1.003, 1.030)
75th-D4	Daytime-only heatwave	1.027 (1.005, 1.050)	1.026 (1.004, 1.050)	1.027 (1.005, 1.050)	1.027 (1.005, 1.050)	1.027 (1.005, 1.050)
	Nighttime-only heatwave	1.014 (0.991, 1.038)	1.007 (0.983, 1.033)	1.014 (0.991, 1.038)	1.014 (0.991, 1.038)	1.014 (0.991, 1.038)
	Compound heatwave	1.017 (1.001, 1.033)	1.015 (0.999, 1.032)	1.017 (1.001, 1.033)	1.017 (1.001, 1.033)	1.017 (1.001, 1.033)
90th-D2	Daytime-only heatwave	1.030 (1.012, 1.048)	1.031 (1.013, 1.050)	1.030 (1.013, 1.048)	1.030 (1.013, 1.048)	1.030 (1.013, 1.048)
	Nighttime-only heatwave	1.014 (0.996, 1.032)	1.006 (0.987, 1.026)	1.014 (0.996, 1.032)	1.014 (0.996, 1.032)	1.014 (0.996, 1.032)
	Compound heatwave	1.027 (1.006, 1.048)	1.025 (1.004, 1.047)	1.027 (1.006, 1.048)	1.027 (1.006, 1.048)	1.027 (1.006, 1.048)
90th-D3	Daytime-only heatwave	1.033 (1.009, 1.057)	1.034 (1.009, 1.059)	1.033 (1.009, 1.057)	1.033 (1.009, 1.057)	1.033 (1.009, 1.057)
	Nighttime-only heatwave	1.015 (0.991, 1.040)	1.017 (0.991, 1.043)	1.015 (0.991, 1.040)	1.015 (0.991, 1.040)	1.015 (0.991, 1.040)
	Compound heatwave	1.026 (1.000, 1.051)	1.023 (0.998, 1.049)	1.026 (1.000, 1.051)	1.026 (1.001, 1.051)	1.026 (1.001, 1.052)
90th-D4	Daytime-only heatwave	1.042 (1.011, 1.075)	1.046 (1.014, 1.079)	1.042 (1.011, 1.075)	1.042 (1.011, 1.075)	1.042 (1.011, 1.075)
	Nighttime-only heatwave	1.012 (0.979, 1.047)	1.001 (0.967, 1.037)	1.012 (0.979, 1.047)	1.012 (0.979, 1.047)	1.012 (0.979, 1.047)
	Compound heatwave	1.037 (1.005, 1.069)	1.035 (1.003, 1.068)	1.037 (1.006, 1.069)	1.037 (1.006, 1.069)	1.037 (1.006, 1.069)

Abbreviations: DF, degrees of freedom. [a]A heat wave was defined as a period equal to or more than two, three, or four consecutive days above the daily threshold. Daytime-only heatwave contains only the 75th or 90th percentile of daily maximum temperature. Nighttime-only heatwave has only the 75th or 90th percentile of daily minimum temperature. Compound heatwave contains 75th or 90th percentile of daily maximum and minimum temperature. The 75th-D2 daytime-only heatwave is a period that only has equal to or more than two consecutive days above the 75th percentile of daily maximum temperature. Others are defined similarly. [b]The main model controlled the long-term trend, seasonality, effects of the day of the week, and time-invariant individual level confounders in design and adjusted for relative humidity and $PM_{2.5}$ using a natural cubic spline with 3 degrees of freedom. [c]The model controlled the long-term trend, seasonality, effects of the day of the week, and time-invariant individual level confounders in design and adjusted for relative humidity, $PM_{2.5}$ and O_3 using a natural cubic spline with 3 degrees of freedom.

© 2023 Guo Y et al. *JAMA Network Open*.

eTable 6. Adjusted Odds Ratios of Preterm Birth Associated With Heat Waves and *P* Values for Effect Modification by Urban, Rural, and Subnational Socioeconomic Regions

	75th-D2[a]								
	**Daytime-only heatwave[a]**			**Nighttime-only heatwave[a]**			**Compound heatwave[a]**		
	**Odds ratio (95% CI)**	**p-value for effect modification**		**Odds ratio (95% CI)**	**p-value for effect modification**		**Odds ratio (95% CI)**	**p-value for effect modification**	
		$P_{z\text{-test}}$[b]	$P_{interaction}$[c]		$P_{z\text{-test}}$[b]	$P_{interaction}$[c]		$P_{z\text{-test}}$[b]	$P_{interaction}$[c]
**Overall**	1.005 (0.991, 1.019)	NA	NA	1.008 (0.994, 1.022)	NA	NA	1.018 (1.005, 1.030)	NA	NA
**Rural/Urban**									
Rural	1.010 (0.981, 1.039)	0.73	0.85	1.025 (0.997, 1.053)	0.17	0.14	1.028 (1.004, 1.053)	0.33	0.35
Urban	1.004 (0.988, 1.020)	[Reference]	[Reference]	1.002 (0.987, 1.018)	[Reference]	[Reference]	1.014 (1.000, 1.028)	[Reference]	
**Subnational Socioeconomic Regions**									
High-income Region	0.991 (0.967, 1.016)	[Reference]	[Reference]	1.003 (0.981, 1.027)	[Reference]	[Reference]	1.003 (0.983, 1.022)	[Reference]	[Reference]
Middle-income Region	1.009 (0.984, 1.034)	0.33	0.25	1.018 (0.994, 1.043)	0.39	0.37	1.031 (1.009, 1.054)	0.06	0.05**
Low-income Region	1.016 (0.993, 1.041)	0.15	0.30	1.004 (0.980, 1.028)	>0.99	0.85	1.024 (1.002, 1.046)	0.16	0.35
**GDP per capita**									
Low	1.015 (0.989, 1.042)	0.38	0.62	1.018 (0.993, 1.044)	0.35	0.26	1.033 (1.010, 1.056)	0.12	0.16
High	1.001 (0.985, 1.018)	[Reference]	[Reference]	1.004 (0.987, 1.020)	[Reference]	[Reference]	1.011 (0.997, 1.026)	[Reference]	[Reference]
**GDP**									
Low	1.009 (0.979, 1.040)	0.74	>0.99	1.019 (0.990, 1.049)	0.41	0.35	1.023 (0.997, 1.049)	0.66	0.72
High	1.004 (0.988, 1.020)	[Reference]	[Reference]	1.005 (0.990, 1.021)	[Reference]	[Reference]	1.016 (1.002, 1.030)	[Reference]	[Reference]
**Population**									
Low	1.000 (0.971, 1.030)	0.73	0.66	1.009 (0.980, 1.039)	0.91	0.91	1.022 (0.996, 1.049)	0.67	0.72
High	1.006 (0.991, 1.022)	[Reference]	[Reference]	1.008 (0.992, 1.023)	[Reference]	[Reference]	1.016 (1.002, 1.030)	[Reference]	[Reference]
**NDVI**									
Low	0.999 (0.982, 1.016)	[Reference]	[Reference]	1.001 (0.984, 1.018)	[Reference]	[Reference]	1.011 (0.996, 1.027)	[Reference]	[Reference]
High	1.018 (0.994, 1.043)	0.20	0.29	1.021 (0.998, 1.045)	0.17	0.12	1.029 (1.009, 1.049)	0.18	0.21

© 2023 Guo Y et al. *JAMA Network Open*.

eTable 6. (Continued)

	75th-D3								
	Daytime-only heatwave			Nighttime-only heatwave			Compound heatwave		
	Odds ratio (95% CI)	p-value for effect modification		Odds ratio (95% CI)	p-value for effect modification		Odds ratio (95% CI)	p-value for effect modification	
		$P_{z\text{-test}}$	$P_{interaction}$		$P_{z\text{-test}}$	$P_{interaction}$		$P_{z\text{-test}}$	$P_{interaction}$
**Overall**	1.013 (0.995, 1.031)	NA	NA	1.017 (0.999, 1.035)	NA	NA	1.016 (1.003, 1.030)	NA	NA
**Rural/Urban**									
Rural	1.050 (1.013, 1.088)	0.02*	0.04*	1.013 (0.978, 1.049)	0.81	0.87	1.030 (1.003, 1.058)	0.25	0.27
Urban	1.002 (0.981, 1.022)	[Reference]	[Reference]	1.018 (0.997, 1.039)	[Reference]	[Reference]	1.011 (0.996, 1.027)	[Reference]	[Reference]
**Subnational Socioeconomic Regions**									
High-income Region	1.013 (0.982, 1.045)	[Reference]	[Reference]	1.019 (0.989, 1.049)	[Reference]	[Reference]	1.005 (0.983, 1.027)	[Reference]	[Reference]
Middle-income Region	1.008 (0.977, 1.040)	0.84	0.97	1.030 (0.998, 1.063)	0.62	0.20	1.025 (1.001, 1.050)	0.22	0.25
Low-income Region	1.019 (0.989, 1.050)	0.77	0.63	1.004 (0.973, 1.037)	0.51	0.56	1.023 (0.998, 1.048)	0.28	0.92
**GDP per capita**									
Low	1.050 (1.016, 1.085)	0.01*	0.03**	1.004 (0.973, 1.037)	0.37	0.48	1.037 (1.012, 1.063)	0.06	0.09
High	0.998 (0.978, 1.019)	[Reference]	[Reference]	1.022 (1.000, 1.044)	[Reference]	[Reference]	1.008 (0.992, 1.024)	[Reference]	[Reference]
**GDP**									
Low	1.054 (1.016, 1.094)	0.02*	0.04**	1.015 (0.978, 1.054)	0.94	0.95	1.034 (1.005, 1.064)	0.17	0.20
High	1.001 (0.981, 1.021)	[Reference]	[Reference]	1.017 (0.996, 1.038)	[Reference]	[Reference]	1.011 (0.996, 1.027)	[Reference]	[Reference]
**Population**									
Low	1.063 (1.025, 1.102)	0.003*	0.006**	1.018 (0.981, 1.057)	0.92	0.85	1.026 (0.996, 1.056)	0.47	0.58
High	0.998 (0.978, 1.019)	[Reference]	[Reference]	1.016 (0.996, 1.037)	[Reference]	[Reference]	1.014 (0.998, 1.029)	[Reference]	[Reference]
**NDVI**									
Low	0.998 (0.976, 1.020)	[Reference]	[Reference]	1.019 (0.997, 1.043)	[Reference]	[Reference]	1.012 (0.995, 1.029)	[Reference]	[Reference]
High	1.043 (1.012, 1.075)	0.02*	0.03**	1.012 (0.982, 1.042)	0.69	0.81	1.025 (1.002, 1.047)	0.39	0.42

© 2023 Guo Y et al. *JAMA Network Open*.

eTable 6. (Continued)

	75th-D4								
	Daytime-only heatwave			Nighttime-only heatwave			Compound heatwave		
	Odds ratio (95% CI)	p-value for effect modification		Odds ratio (95% CI)	p-value for effect modification		Odds ratio (95% CI)	p-value for effect modification	
		$P_{z\text{-test}}$	$P_{interaction}$		$P_{z\text{-test}}$	$P_{interaction}$		$P_{z\text{-test}}$	$P_{interaction}$
**Overall**	1.027 (1.005, 1.050)	NA	NA	1.014 (0.991, 1.038)	NA	NA	1.017 (1.001, 1.033)	NA	NA
**Rural/Urban**									
Rural	1.059 (1.014, 1.107)	0.11	0.15	0.994 (0.950, 1.040)	0.31	0.35	1.016 (0.985, 1.048)	0.96	0.92
Urban	1.017 (0.992, 1.043)	[Reference]	[Reference]	1.021 (0.994, 1.049)	[Reference]	[Reference]	1.017 (0.999, 1.035)	[Reference]	[Reference]
**Subnational Socioeconomic Regions**									
High-income Region	1.038 (1.000, 1.078)	[Reference]	[Reference]	0.992 (0.954, 1.030)	[Reference]	[Reference]	1.006 (0.981, 1.031)	[Reference]	[Reference]
Middle-income Region	1.001 (0.963, 1.040)	0.18	0.25	1.039 (0.997, 1.083)	0.10	0.11	1.022 (0.994, 1.051)	0.42	0.40
Low-income Region	1.042 (1.005, 1.081)	0.88	0.92	1.020 (0.979, 1.063)	0.32	0.24	1.028 (1.000, 1.058)	0.26	0.51
**GDP per capita**									
Low	1.061 (1.020, 1.104)	0.06	0.10	0.981 (0.942, 1.023)	0.06	0.10	1.039 (1.010, 1.069)	0.08	0.12
High	1.014 (0.988, 1.040)	[Reference]	[Reference]	1.029 (1.001, 1.058)	[Reference]	[Reference]	1.008 (0.990, 1.027)	[Reference]	[Reference]
**GDP**									
Low	1.064 (1.017, 1.113)	0.09	0.15	0.992 (0.945, 1.041)	0.31	0.37	1.045 (1.012, 1.079)	0.06	0.08
High	1.017 (0.992, 1.043)	[Reference]	[Reference]	1.021 (0.994, 1.048)	[Reference]	[Reference]	1.009 (0.991, 1.027)	[Reference]	[Reference]
**Population**									
Low	1.079 (1.032, 1.128)	0.02*	0.03**	1.028 (0.980, 1.080)	0.51	0.47	1.028 (0.994, 1.063)	0.47	0.58
High	1.012 (0.988, 1.038)	[Reference]	[Reference]	1.010 (0.983, 1.036)	[Reference]	[Reference]	1.014 (0.996, 1.032)	[Reference]	[Reference]
**NDVI**									
Low	1.015 (0.988, 1.043)	[Reference]	[Reference]	1.019 (0.990, 1.050)	[Reference]	[Reference]	1.013 (0.993, 1.033)	[Reference]	[Reference]
High	1.051 (1.013, 1.091)	0.13	0.18	1.005 (0.968, 1.044)	0.57	0.67	1.024 (0.999, 1.050)	0.49	0.52

© 2023 Guo Y et al. *JAMA Network Open*.

eTable 6. (Continued)

	90th-D2								
	Daytime-only heatwave			Nighttime-only heatwave			Compound heatwave		
	Odds ratio (95% CI)	p-value for effect modification		Odds ratio (95% CI)	p-value for effect modification		Odds ratio (95% CI)	p-value for effect modification	
		$P_{z\text{-test}}$	$P_{interaction}$		$P_{z\text{-test}}$	$P_{interaction}$		$P_{z\text{-test}}$	$P_{interaction}$
**Overall**	1.030 (1.012, 1.048)	NA	NA	1.014 (0.996, 1.032)	NA	NA	1.027 (1.006, 1.048)	NA	NA
**Rural/Urban**									
Rural	1.038 (1.001, 1.076)	0.65	0.75	1.020 (0.985, 1.056)	0.71	0.69	1.030 (0.989, 1.072)	0.86	0.89
Urban	1.028 (1.008, 1.049)	[Reference]		1.012 (0.991, 1.033)	[Reference]	[Reference]	1.025 (1.001, 1.050)	[Reference]	[Reference]
**Subnational Socioeconomic Regions**									
High-income Region	1.023 (0.992, 1.056)	[Reference]	[Reference]	1.018 (0.989, 1.048)	[Reference]	[Reference]	1.034 (1.001, 1.068)	[Reference]	[Reference]
Middle-income Region	1.053 (1.021, 1.086)	0.19	0.14	1.026 (0.993, 1.059)	0.74	0.72	1.029 (0.992, 1.067)	0.85	0.89
Low-income Region	1.019 (0.990, 1.048)	0.84	0.64	1.000 (0.969, 1.033)	0.42	0.42	1.018 (0.980, 1.057)	0.54	0.34
**GDP per capita**									
Low	1.046 (1.012, 1.081)	0.30	0.45	1.006 (0.974, 1.040)	0.60	0.64	1.053 (1.014, 1.093)	0.13	0.15
High	1.024 (1.004, 1.045)	[Reference]	[Reference]	1.017 (0.995, 1.039)	[Reference]	[Reference]	1.017 (0.992, 1.042)	[Reference]	[Reference]
**GDP**									
Low	1.019 (0.981, 1.057)	0.50	0.37	0.977 (0.941, 1.015)	0.03*	0.04**	1.059 (1.014, 1.106)	0.12	0.13
High	1.033 (1.013, 1.054)	[Reference]	[Reference]	1.025 (1.004, 1.046)	[Reference]	[Reference]	1.018 (0.995, 1.042)	[Reference]	[Reference]
**Population**									
Low	1.014 (0.977, 1.052)	0.32	0.28	0.984 (0.947, 1.023)	0.10	0.11	1.011 (0.966, 1.058)	0.44	0.34
High	1.035 (1.015, 1.056)	[Reference]	[Reference]	1.022 (1.001, 1.043)	[Reference]	[Reference]	1.031 (1.008, 1.055)	[Reference]	[Reference]
**NDVI**									
Low	1.024 (1.002, 1.046)	[Reference]	[Reference]	1.012 (0.990, 1.035)	[Reference]	[Reference]	1.016 (0.990, 1.043)	[Reference]	[Reference]
High	1.044 (1.013, 1.075)	0.30	0.38	1.017 (0.987, 1.047)	0.83	0.79	1.046 (1.012, 1.081)	0.17	0.18

© 2023 Guo Y et al. *JAMA Network Open*.

eTable 6. (Continued)

	90th-D3								
	Daytime-only heatwave			Nighttime-only heatwave			Compound heatwave		
	Odds ratio (95% CI)	p-value for effect modification		Odds ratio (95% CI)	p-value for effect modification		Odds ratio (95% CI)	p-value for effect modification	
		$P_{z\text{-test}}$	$P_{interaction}$		$P_{z\text{-test}}$	$P_{interaction}$		$P_{z\text{-test}}$	$P_{interaction}$
**Overall**	1.033 (1.009, 1.057)	NA	NA	1.015 (0.991, 1.040)	NA	NA	1.026 (1.000, 1.051)	NA	NA
**Rural/Urban**									
Rural	1.091 (1.042, 1.143)	0.01*	0.01**	1.037 (0.991, 1.086)	0.28	0.27	1.023 (0.974, 1.076)	0.94	0.93
Urban	1.014 (0.987, 1.041)	[Reference]	[Reference]	1.007 (0.978, 1.036)	[Reference]	[Reference]	1.026 (0.997, 1.056)	[Reference]	[Reference]
**Subnational Socioeconomic Regions**									
High-income Region	1.027 (0.984, 1.072)	[Reference]	[Reference]	1.026 (0.987, 1.066)	[Reference]	[Reference]	1.005 (0.966, 1.046)	[Reference]	[Reference]
Middle-income Region	1.021 (0.978, 1.066)	0.84	>0.99	1.018 (0.973, 1.066)	0.81	0.84	1.070 (1.025, 1.117)	0.03	0.04**
Low-income Region	1.047 (1.010, 1.086)	0.50	0.68	1.004 (0.960, 1.049)	0.46	0.46	1.007 (0.961, 1.056)	0.94	0.87
**GDP per capita**									
Low	1.069 (1.024, 1.115)	0.06	0.11	0.996 (0.954, 1.040)	0.30	0.32	1.036 (0.989, 1.085)	0.63	0.65
High	1.018 (0.990, 1.047)	[Reference]	[Reference]	1.024 (0.994, 1.055)	[Reference]	[Reference]	1.022 (0.992, 1.052)	[Reference]	[Reference]
**GDP**									
Low	1.068 (1.018, 1.120)	0.12	0.19	1.005 (0.956, 1.056)	0.65	0.67	1.023 (0.970, 1.079)	0.91	0.91
High	1.022 (0.995, 1.050)	[Reference]	[Reference]	1.018 (0.990, 1.047)	[Reference]	[Reference]	1.027 (0.998, 1.056)	[Reference]	[Reference]
**Population**									
Low	1.070 (1.020, 1.121)	0.10	0.13	0.994 (0.945, 1.047)	0.38	0.40	1.041 (0.985, 1.100)	0.57	0.62
High	1.021 (0.994, 1.049)	[Reference]	[Reference]	1.021 (0.993, 1.050)	[Reference]	[Reference]	1.022 (0.994, 1.051)	[Reference]	[Reference]
**NDVI**									
Low	1.007 (0.978, 1.037)	[Reference]	[Reference]	1.009 (0.978, 1.042)	[Reference]	[Reference]	1.035 (1.003, 1.068)	[Reference]	[Reference]
High	1.079 (1.039, 1.122)	0.005*	0.008**	1.024 (0.985, 1.065)	0.56	0.53	1.011 (0.971, 1.053)	0.38	0.38

© 2023 Guo Y et al. *JAMA Network Open*.

eTable 6. (Continued)

	90th-D4								
	Daytime-only heatwave			Nighttime-only heatwave			Compound heatwave		
	Odds ratio (95% CI)	p-value for effect modification		Odds ratio (95% CI)	p-value for effect modification		Odds ratio (95% CI)	p-value for effect modification	
		$P_{z\text{-test}}$	$P_{interaction}$		$P_{z\text{-test}}$	$P_{interaction}$		$P_{z\text{-test}}$	$P_{interaction}$
**Overall**	1.042 (1.011, 1.075)	NA	NA	1.012 (0.979, 1.047)	NA	NA	1.037 (1.005, 1.069)	NA	NA
**Rural/Urban**									
Rural	1.093 (1.028, 1.161)	0.08	0.10	1.033 (0.968, 1.101)	0.49	0.48	1.031 (0.969, 1.098)	0.85	0.84
Urban	1.026 (0.991, 1.063)	[Reference]	[Reference]	1.005 (0.966, 1.046)	[Reference]	[Reference]	1.038 (1.002, 1.075)	[Reference]	[Reference]
**Subnational Socioeconomic Regions**									
High-income Region	1.049 (0.992, 1.110)	[Reference]	[Reference]	1.030 (0.978, 1.085)	[Reference]	[Reference]	1.024 (0.975, 1.076)	[Reference]	[Reference]
Middle-income Region	1.037 (0.981, 1.096)	0.76	0.89	1.008 (0.945, 1.075)	0.60	0.62	1.051 (0.997, 1.108)	0.48	0.52
Low-income Region	1.043 (0.994, 1.094)	0.87	0.75	1.000 (0.941, 1.063)	0.47	0.49	1.044 (0.984, 1.107)	0.63	0.83
**GDP per capita**									
Low	1.066 (1.007, 1.127)	0.36	0.48	0.992 (0.935, 1.053)	0.42	0.44	1.066 (1.006, 1.129)	0.27	0.29
High	1.033 (0.996, 1.071)	[Reference]	[Reference]	1.022 (0.981, 1.065)	[Reference]	[Reference]	1.026 (0.990, 1.064)	[Reference]	[Reference]
**GDP**									
Low	1.073 (1.008, 1.141)	0.30	0.42	0.991 (0.926, 1.060)	0.48	0.49	1.061 (0.995, 1.133)	0.43	0.43
High	1.033 (0.998, 1.070)	[Reference]	[Reference]	1.019 (0.981, 1.060)	[Reference]	[Reference]	1.030 (0.995, 1.066)	[Reference]	[Reference]
**Population**									
Low	1.077 (1.013, 1.145)	0.23	0.28	0.978 (0.910, 1.050)	0.28	0.29	1.070 (0.998, 1.146)	0.33	0.40
High	1.031 (0.996, 1.068)	[Reference]	[Reference]	1.022 (0.984, 1.062)	[Reference]	[Reference]	1.029 (0.995, 1.065)	[Reference]	[Reference]
**NDVI**									
Low	1.024 (0.985, 1.064)	[Reference]	[Reference]	1.009 (0.967, 1.054)	[Reference]	[Reference]	1.047 (1.007, 1.089)	[Reference]	[Reference]
High	1.077 (1.024, 1.132)	0.12	0.15	1.017 (0.965, 1.073)	0.82	0.79	1.022 (0.973, 1.074)	0.46	0.46

NA denotes not applicable. Abbreviations: CI, confidence interval. Models controlled the long-term trend, seasonality, effects of the day of the week, and time-invariant individual level confounders in design and adjusted for relative humidity and $PM_{2.5}$ using a natural cubic spline with 3 degrees of freedom. [a]A heat wave was defined as a period equal to or more than two, three, or four consecutive days above the daily threshold. Daytime-only heatwave contains only the 75th or 90th percentile of daily maximum temperature. Nighttime-only heatwave has only the 75th or 90th percentile of daily minimum temperature. Compound heatwave contains 75th or 90th percentile of daily maximum and minimum temperature. The 75th-D2 daytime-only heatwave is a period that only has equal to or more than two consecutive days above the 75th percentile of daily maximum temperature. Others are defined similarly. [b]To determine whether the risk estimates are statistically different, for example, rural vs. urban, we have $Z = \frac{\beta_{urban} - \beta_{rural}}{\sqrt{se(\beta_{urban})^2 + se(\beta_{rural})^2}}$. *p<0.05 for two samples and 0.025 for triple-group comparisons. [c]We tested the significance of interaction terms of heatwave exposure variable and urban-rural or subnational socioeconomic regions' category variable in models. ** p < 0.05

© 2023 Guo Y et al. *JAMA Network Open*.

eReferences

1. Deng K, Liang J, Mu Y, et al. Preterm births in China between 2012 and 2018: an observational study of more than 9 million women. *Lancet Glob Health.* 2021;9(9):e1226-e1241.
2. Liang J, Mu Y, Li X, et al. Relaxation of the one child policy and trends in caesarean section rates and birth outcomes in China between 2012 and 2016: observational study of nearly seven million health facility births. *Bmj.* 2018;360:k817.
3. Mu Y, Wang X, Li X, et al. The national maternal near miss surveillance in China: A facility-based surveillance system covered 30 provinces. *Medicine (Baltimore).* 2019;98(44):e17679.
4. Ministry of Health C. *National Health Statistical Yearbook.* Beijing: China Union Medical College Press; 2010.
5. Wang J, Yeh AG. Administrative restructuring and urban development in China: Effects of urban administrative level upgrading. *Urban Studies.* 2020;57:1201-1223.
6. 1km Gridded Population Dataset of China. Data registration and Publication System of Data Center for Resources and Environmental Sciences, CAS; 2017. http://www.resdc.cn/DOI. Accessed September 25, 2021.
7. 1km Gridded GDP dataset of China. Data registration and Publication System of Data Center for Resources and Environmental Sciences, CAS; 2017.
8. 1km Monthly NDVI Spatial Distribution Dataset of China. Data registration and Publication System of Data Center for Resources and Environmental Sciences, CAS; 2018. http://www.resdc.cn/DOI. Accessed November 20, 2022
9. Li L, Wu J, Pu D, et al. Factors associated with the age of natural menopause and menopausal symptoms in Chinese women. *Maturitas.* 2012;73(4):354-360.
10. Song Y, Ma J, Hu P, Zhang B. Geographic distribution and secular trend of menarche in 9-18 year-old Chinese Han girls [in Chinese]. *Journal of Peking University(Health Sciences).* 2011;43(03):360-364.
11. Strand LB, Barnett AG, Tong S. Methodological challenges when estimating the effects of season and seasonal exposures on birth outcomes. *BMC Med Res Methodol.* 2011;11:49.
12. Capital Institute of Pediatrics, The Coordinating Study Group of Nine Cities on the Physical Growth and Development of Children. Growth standard curves of birth weight, length and head circumference of Chinese newborns of different gestation[in Chinese]. *Chinese Journal of Pediatrics.* 2020;58(09):738-746.

© 2023 Guo Y et al. *JAMA Network Open*.
