# Supplementary material for: Association of Daytime-Only, Nighttime-Only, and Compound Heat Waves With Preterm Birth by Urban-Rural Area and Regional Socioeconomic Status in China
Source: JAMA Netw Open. 2023 Aug 11;6(8):e2326987. doi: 10.1001/jamanetworkopen.2023.26987 (PMC10422195; doi:10.1001/jamanetworkopen.2023.26987)
Supplement: Supplement 2. — Data Sharing Statement [file jamanetwopen-e2326987-s002.pdf]

Data Sharing Statement

Guo. Association of Daytime-Only, Nighttime-Only, and Compound Heat Waves With Preterm Birth by Urban-Rural Area and Regional Socioeconomic Status in China. *JAMA Netw Open*. Published August 11, 2023. doi:10.1001/jamanetworkopen.2023.26987

Data
Data available: No

Additional Information
Explanation for why data not available: The NMNMSS data used in this study were collected under a data-sharing agreement and cannot be made publicly available but are available on request from the corresponding authors on reasonable request and with the permission of the National Health Commission of China.
